# Fibrotic lung ECM upregulates SDC4/integrin-αvβ1 interaction and the interfering peptide SDC4_87-131_ and its derivative peptides alleviate pulmonary fibrosis

**DOI:** 10.1093/rb/rbaf057

**Published:** 2025-06-16

**Authors:** Lihua Zhu, Lingfeng Xie, Yupeng Zhi, Yihao Huang, Hongkui Chen, Zibin Chen, Jinsheng Hong, Yansong Guo, Chun Chen

**Affiliations:** School of Pharmacy, Fujian Medical University, Fuzhou, Fujian 350122, China; School of Pharmacy, Fujian Medical University, Fuzhou, Fujian 350122, China; School of Pharmacy, Fujian Medical University, Fuzhou, Fujian 350122, China; School of Pharmacy, Fujian Medical University, Fuzhou, Fujian 350122, China; Department of Cardiology, Shengli Clinical Medical College of Fujian Medical University, Fujian Provincial Hospital, Fuzhou University Affiliated Provincial Hospital, Fuzhou, Fujian 350001, China; School of Pharmacy, Fujian Medical University, Fuzhou, Fujian 350122, China; Department of Radiotherapy, Cancer Center, The First Affiliated Hospital of Fujian Medical University, Fuzhou, Fujian 350005, China; Department of Cardiology, Shengli Clinical Medical College of Fujian Medical University, Fujian Provincial Hospital, Fuzhou University Affiliated Provincial Hospital, Fuzhou, Fujian 350001, China; School of Pharmacy, Fujian Medical University, Fuzhou, Fujian 350122, China; Fujian Key Laboratory of Natural Medicine Pharmacology, Fujian Medical University, Fuzhou, Fujian 350122, China

**Keywords:** pulmonary fibrosis, decellularized extracellular matrix, fibroblast activation, SDC4, integrin, interfering peptide

## Abstract

Fibroblast activation promotes remodeling of the extracellular matrix (ECM), and the fibrotic remodeling ECM further stimulating fibroblast activation and advancing pulmonary fibrosis (PF). syndecan-4 (SDC4) is the key mediator of ECM-cell signaling, but its action in PF remains unclear. Using decellularized lung ECM (dECM), this study found that fibrotic ECM enhanced fibroblast activation via SDC4-regulated integrin-αvβ1 expression and activation, and FAK/AKT phosphorylation. Meanwhile, SDC4 knockdown inhibited fibrotic ECM-induced TGF-β1 synthesis and PKCα activation. A Duolink-proximity ligation assay confirmed extracellular interactions between SDC4 and integrin-αvβ1, and the SDC4 blocking antibody Anti-SDC4_(93-121)_ prevented this interaction, resulting in an effect consistent with knockdown of SDC4. The interfering peptide SDC4_87-131_ diminished the interaction between SDC4 and integrin-αvβ1, subsequently inhibited the activation of FAK/AKT, Smad2/3 and PKCα/NF-κB pathways and exhibited anti-PF activity comparable to that of SDC4 knockdown and Anti-SDC4_(93-121)_. A docking mode of SDC4_87-131_ with the Calf-1/Calf-2 domain of integrin-αv was constructed by using the AlphaFold2-Multimer model, and peptide design was performed to obtain a novel polypeptide chain CS-9 with enhanced anti-PF effect. This study found that the biomaterial, lung ECM, regulates fibroblast activation through the collaboration of SDC4 and integrin-αvβ1, and obtained a novel SDC4_87-131_-derived peptide that may prevent fibrotic ECM from promoting PF.

## Graphical abstract

**Figure:**
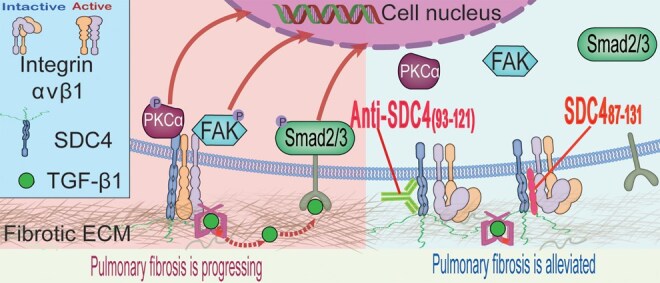

## Introduction

Pulmonary fibrosis (PF) represents the terminal stage of various interstitial lung diseases, characterized by compromised alveolar gas exchange, potentially leading to respiratory failure and death [1, 2]. Among these, idiopathic PF (IPF) is noted for its unrelenting progression and dire prognosis. Other interstitial lung diseases such as hylactic pneumonia, silicosis and rheumatoid arthritis possess a 30–50% likelihood of advancing to PF [1]. Research indicates that 25–47% of patients with COVID-19-related lung disease develop impaired gas exchange and PF [3–5]. Pirfenidone and nintedanib are the sole anti-PF medications approved for IPF, with their effectiveness on PF stemming from other causes yet to be assessed, as per 2022 International Guidelines [6]. Thus, identifying new targets and treatments for PF is crucial for its prevention and management.

Despite varying causes, a shared pathological response in PF is an excessive wound-healing process. Fibroblast undergo prolific proliferation and activation, leading to abundant matrix protein deposition and extracellular matrix (ECM) remodeling and stiffening [1, 7]. Studies have shown that hardened ECM can amplify mechanotransduction pathways, including integrin β1, focal adhesion kinase (FAK), Rho-associated protein kinase (ROCK) [8, 9]. These pathways drive the expression of profibrotic genes such as α-smooth muscle actin (α-SMA) and type I collagen, culminating in the activation of fibroblasts and increased contractility [8, 10]. Contractile fibroblasts facilitate the release of transforming growth factor-β1 (TGF-β1) from its latent state bound to latency-associated peptide (LAP) through RGD-containing integrins (αvβ1/αvβ6/αvβ3/αvβ5) [10], amplifying the fibrotic response through the TGF-β/Smad2/3 signaling pathway [11, 12]. These findings suggest that fibrotic ECM remodeling, fibroblast activation and activation of the TGF-β signaling pathway establish a positive feedback loop, driving the continuous progression of PF [13].

The intact mechanotransduction of the ECM necessitates the concerted action of integrins and syndecan-4 (SDC4) [14]. Syndecans (SDCs) represent a class of transmembrane proteoglycan receptors comprising SDC-1/-2/-3/-4 [15]. The extracellular region of SDCs possesses sugar chains that bind to the heparin-binding domain of ECM component proteins, while the intracellular region connects to actin, thus, contributing to biomechanical signaling [16]. Notably, SDC4 is the sole member of the SDC family involved in integrin-mediated FA formation [17]. Integrins mechanically connect the cytoskeleton to the ECM, forming focal adhesions (FAs), thus, playing a central role in cell adhesion, migration and polarization [18]. The intracellular variable region of SDC4 recruits and activates PKCα. PKCα releases Rho from its substrate RhoGDIα, subsequently participating in FA formation and the phosphorylation activation of FAK [17]. These findings suggest that SDC4 may participate in the promotion of fibrotic ECM to PF by affecting the integrin/FAK pathway, but there is no relevant research yet.

Apart from mediating the transmission of biomechanical forces, the activation of PKCα, recruited by SDC4, can enhance the activities of extracellular signal-regulated kinase 1/2 (ERK1/2) and nuclear factor kappa B (NF-κB), participating in cell proliferation, activation and the secretion of inflammatory factors [19, 20]. In experimental asthma and rheumatoid arthritis, knocking down SDC4 can inhibit the inflammatory response and relieve the symptoms [21, 22]. These findings underscore that SDC4 also mediates inflammatory responses, with anti-inflammation serving as another pivotal strategy against PF [23, 24].

The fibrotic lung ECM plays a critical role in promoting fibroblast activation and expediting the progression of PF. Investigating its mechanisms will reveal new targets for combating PF and aid in designing biomaterials that more effectively replicate the normal lung microenvironment. Through *in vitro* and *in vivo* experiments, this study sought to elucidate the role and mechanisms of the fibrotic ECM in promoting pulmonary fibrosis via SDC4, assess the anti-PF effects of the interfering peptide SDC4_87-131_, evaluate its impact on the interaction between SDC4 and integrin-αvβ1, and refine SDC4_87-131_ to derive a new polypeptide with enhanced anti-pulmonary fibrosis effects.

## Materials and methods

### dECM stimulated fibroblast

Decellularized lung ECM (dECM) was prepared as described in previous study [25]. 2 × 10^5^ cells/mL NIH3T3 cells (mouse embryonic lung fibroblasts, purchased from the Stem Cell Bank of the Chinese Academy of Sciences, Shanghai, China) were seeded on the dECM slices attached to the bottom of the culture dish and cultured in DMEM medium containing 0.5% fetal bovine serum (Gibco, Waltham, MA, USA) at 37°C and 5% CO_2_.

### Sample preparation and liquid chromatography-tandem mass spectrometry (LC-MS/MS) analysis

NIH3T3 cells induced by normal lung ECM (dECM-Nor) or fibrotic remodeled lung ECM (dECM-PF) were collected, and protein extraction and concentration determination were performed. After routine trypsin digestion, the obtained peptides were desalted and then separated using a reversed-phase analytical column (25 cm length, 100 μm i.d.) on a nanoElute UHPLC system (Bruker Daltonics) with a constant flow rate gradient elution of 450 nL/min. The peptides were then subjected to MS/MS analysis, database search and bioinformatics analysis. The specific method was described in the Supplementary Methods. A two-way ANOVA determined significance between groups. Proteins with a fold change > 1.2 and a *P* values < 0.05 were considered significant.

The mass spectrometry proteomics data have been deposited to the ProteomeXchange Consortium (http://proteomecentral.proteomexchange.org) via the iProX partner repository with the dataset identifier PXD042526.

### Animal experiments

C57BL/6J mice (male, 8-to 9-week old, 18–22 g, SPF grade) were purchased from Shanghai Slake Laboratory Animal Co., Ltd. [SCXK (Hu) 2022-0004, Shanghai, China]. Studies have reported that C57BL/6J male mice exhibit significantly higher sensitivity to BLM PF than female mice [26, 27], so we chose a single gender (male) to avoid the influence of this variable. The mice were housed in an SPF environment with 22–25°C, 46–65% humidity and 12 h/12 h light-dark alternation, with free access to water and food. This animal experiment was approved by the Experimental Animal Welfare Ethics Committee of Fujian Medical University (No. FJMU IACUC 2021-0465), and was carried out in accordance with the National Research Council's Guide for the Care and Use of Laboratory Animals.

After the mice were anesthetized (1% sodium pentobarbital 50 mg/kg was injected intraperitoneally), 150 μL of bleomycin (BLM, 5 mg/mL, BIO-000001) was inhaled into the lungs of the mice by tracheal inhalation instillation. One day later, the mice were randomly divided into model and drug treatment groups, and the mice in the control group were only subjected to saline inhalation.

### Duolink^®^ proximity ligation assay

Cells or tissue slices were attached to slides, fixed and permeabilized and blocked with Duolink^TM^ (Merck, MO, USA) blocking buffer. Antibodies against the extracellular domain of SDC4 (5G9, mouse IgG, SCBT #sc-12766), integrin-αv (rabbit IgG, CST #4711) and integrin-β1 (rabbit IgG, CST #34971) were diluted and added to the samples and incubated overnight at 4°C. After washing, PLUS and MINUS (proximity ligation assay, PLA) probes were added to the samples and incubated at 37°C for 1 h. After washing, the samples were sequentially incubated with PLUS and MINUS (PLA) probes, ligation buffer and amplification buffer containing polymerase. Finally, the samples were mounted with *in situ* mounting medium containing DAPI. Images were acquired using a Leica SP5 confocal microscope (Leica Biosystems, Germany) and quantitative analysis was performed using ImageJ software.

### Preparation of polyclonal antibody of Anti-SDC4_(93-121)_

According to the reference method [22], the peptide containing the SDC4 extracellular region (N93-V121) sequence (NAQPGIRVPSEPKELEENEVIPKRAPSDV) was synthesized, Cys was added to the N-terminus and it was coupled to Keyhole Limpet Hemocyanin (KLH) to prepare the antigen. Rabbit polyclonal antibody was prepared by Shanghai Nuoyou Biotechnology Co., Ltd. (Shanghai, China). The antigen solution was injected into New Zealand rabbits routinely to stimulate the immune response. Polyclonal antibodies were purified from serum using protein-A affinity chromatography and the purified antibodies were identified using enzyme-linked immunosorbent assay (ELISA).

### AlphaFold2-multimer was used to construct a protein-peptide binding pattern and SDC4_87-131_ was modified and designed based on the binding pattern

The AlphaFold2-multimer protein interaction prediction model was used [28]. After inputting the integrin-αv and SDC4_87-131_ sequences, homologous sequences were searched in different protein sequence databases (BFD, Uniref90, Uniprot and Mgnify, etc.). The homologous sequences of the two sequences were then spliced by comparing the species sources. Based on the spliced homologous sequences, the structure was predicted by protein language encoding and subsequent loop structure prediction modules. Then, the main chain-fixed protein structure energy was minimized and optimized to obtain multiple predicted binding modes. Each binding mode was given a confidence score by the algorithm (i.e. the predicted distance-based protein local structure score plDDT). We simulated the SDC4_87-131_/integrin-αv complex with an explicit water model using all-atom molecular dynamics simulations, and used the standard MMPBSA/GBSA calculation tool included with AmberTools18 (2018 release) to calculate the binding free energy, with the average value as the final result. We then optimized the SDC4_87-131_ peptide chain (45 aa) based on the key binding sites: small non-charged amino acid fragments far away from the key sites were removed, the contact between the positively charged fragments and the corresponding negatively charged fragments was enhanced, and the α-helix region near the key docking site on αv was replaced into the modified peptide (since the α-helix is prone to dimerization). The Protein MPNN algorithm was used for docking energy calculation as an initial screening, and modified peptides with docking energies like that of SDC4_87-131_ (MMGBSA/MMGPSA) and a shortened sequence (30–40 aa) were selected.

### Surface plasmon resonance

The Biacore 8K+ system (GE Healthcare) was used at 25°C. The ligand integrin-αvβ1 (1 mg/mL) was routinely coupled to the sensor chip (Series S Sensor Chip CM5, Cytiva, #100530). The peptide samples SDC4_87-131_ and CS-1, -2, -3, -4, -8, -9, -10 were dissolved in PBS, and then, the samples with different concentration gradients (0.78, 1.56, 3.125, 6.25, 12.5, 25, 50, 100 μM) were injected, respectively, under the driving of buffer PBST (PBS + 0.05% Tween-20). The signal intensity was quantified in relative units (RU), and binding affinity was determined using the dissociation constant (*K*_*d*_). After the experiment, 10 mM glycine-HCl buffer solution (pH = 2) was selected to regenerate the chip surface.

Additional methods are provided in the Supplementary Methods.

### Statistical analysis

Statistical analyses were performed using GraphPad Prism, and the data were expressed as $\left(\bar{x}\pm s\right)$. Normality distribution was assessed using the Kolmogorov–Smirnov test. Two-tailed unpaired *t*-tests, one-way ANOVA with Dunnett’s post-test or two-way ANOVA with Tukey tests were used to analyze data with a normal distribution. Data with a non-normal distribution were analyzed using Wilcoxon rank-sum tests and Kruskal–Wallis tests with Dunn’s test. *P* < 0.05 was considered as statistically significant. The investigator responsible for data analysis blinded to which samples represent control and treatment groups.

## Results

### SDC4 is persistently overexpressed in PF and contributes to fibroblast activation induced by fibrotic ECM

**Figure 1.**
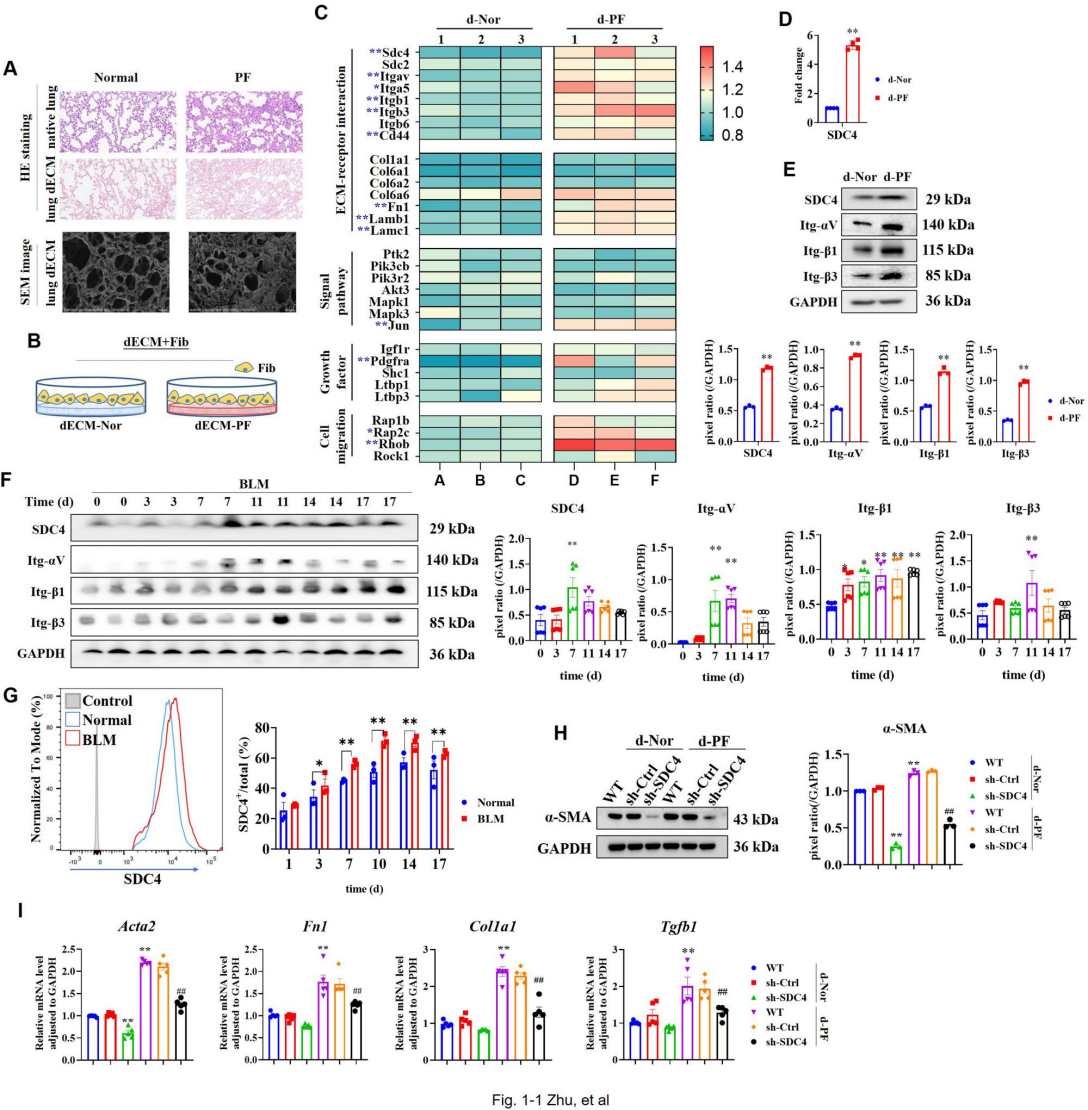
High expression of SDC4 was observed in PF both *in vitro* and *in viv*o, and SDC4 knockdown inhibited fibroblast activation. (**A**) Normal and fibrotic lung tissues were decellularized, and HE staining was used to observe the lung ECM scaffolds after decellularization. Transmission electron microscopy was used to observe the microstructure of the ECM scaffolds. (**B**) Schematic diagram of fibroblast seeded on dECMs (dECM+Fib culture). (**C**) NIH3T3 cells were seeded on dECM-Nor (d-Nor) and dECM-PF (d-PF) and cultured for 48 hr. Cells were harvested for proteomic analysis, and the differential expression of proteins was displayed in a heatmap (*n* = 3). Note: * *P* value < 0.05 and fold change > 1.2, **P < 0.01 and fold change >1.2. (**D**) RT-PCR was used to detect the mRNA levels of SDC4 (*n* = 4). (**E**) Protein expression levels were measured by Western blot, and pixel ratios were analyzed using ImageJ (*n* = 3). Note: *P < 0.05, **P < 0.01 vs. d-Nor group. (**F**, **G**) Mice inhaled BLM via the oropharynx, and lung tissues were collected before inhalation and on days 3, 7, 11, 14 and 17 post-inhalations. (**F**) Protein expression levels in lung tissues were determined (*n* = 2). Note: *P < 0.05, **P < 0.01 vs. 0 day. (**G**) Flow cytometry analyzed the expression levels of SDC4 in lung tissues (*n* = 3). Note: *P < 0.05, **P < 0.01. (**H**) Following SDC4 knockdown, α-SMA expression was assessed (*n* = 3). (**I**) RT-PCR measured mRNA levels of α-SMA (*Acta2*), FN (*Fn1*), Collagen I (*Col1a1*) and TGF-β1 (*Tgfb1*) post-SDC4 knockdown (*n* = 5). Note: *P < 0.05, **P < 0.01 vs. WT in d-Nor group; #*P* < 0.05, ##*P* < 0.01 vs. WT in d-PF group. d-Nor: dECM-Nor, d-PF: dECM-PF.

Decellularized normal and fibrotic lung tissues were prepared. Hematoxylin and eosin (HE) staining confirmed the absence of cell residue post-treatment, retaining only the lung tissue scaffold (Figure 1A and Supplementary Figure S1A). Scanning electron microscopy showed that the alveolar septa of the decellularized fibrotic lung ECM (dECM-PF) were thickened and irregular (Figure 1A). Fibroblast was seeded on dECM to simulate the lung environment (dECM+Fib, Figure 1B and Supplementary Figure S1B), and cells were collected for proteomic analysis. The results showed that compared with dECM-Nor, dECM-PF induced upregulation of 305 proteins and downregulation of 98 proteins in fibroblast (Supplementary Figure S1C). Among these, increased expression was noted for fibroblast activation markers PDGFRα and fibronectin (FN), along with SDC4, integrin-αv, -β1, -β3 and RhoB (Figure 1C). Functional enrichment analysis highlighted the upregulation of glycoprotein biosynthetic processes, ECM-receptor interactions and FN binding (Supplementary Figure S1D). Both RT-PCR and WB verified that dECM-PF stimulated the upregulation of SDC4 and integrin-αv, -β1 and -β3 (Figure 1D and E). Single-cell sequencing datasets of IPF (GSE213017) [29], COVID-19 (GSE227136 + GSE149878) [30, 31], radiation-induced PF in mice (GSE211713) [32] and BLM-induced PF in mice (GSE210341) [33] revealed high levels of SDC4 mRNA in fibroblast cells (Supplementary Figure S2A–D). In our study, SDC4 expression in lung tissues of BLM-induced PF significantly increased from day 7 to day 17, along with variable increases in integrin-αv, -β1 and -β3 (Figure 1F). Flow cytometry detected dynamic SDC4 expression in lung tissues of BLM-induced PF, producing similar results (Figure 1G).

Knockdown of SDC4 in fibroblast using sh-RNA (Supplementary Figure S3A and B) curtailed cell proliferation (Supplementary Figure S3C). In the culture of dECM+Fib, knockdown of SDC4 downregulated the expression levels of fibroblast activation markers α-SMA, FN, Collagen I and TGF-β1 (Supplementary Figure S3D and  Figure 1H and I), indicating that SDC4 knockdown blocked dECM-PF-induced fibroblast activation.

### Knockdown of SDC4 in lung tissue can alleviate PF and reduce fibroblast activation

**Figure 2.**
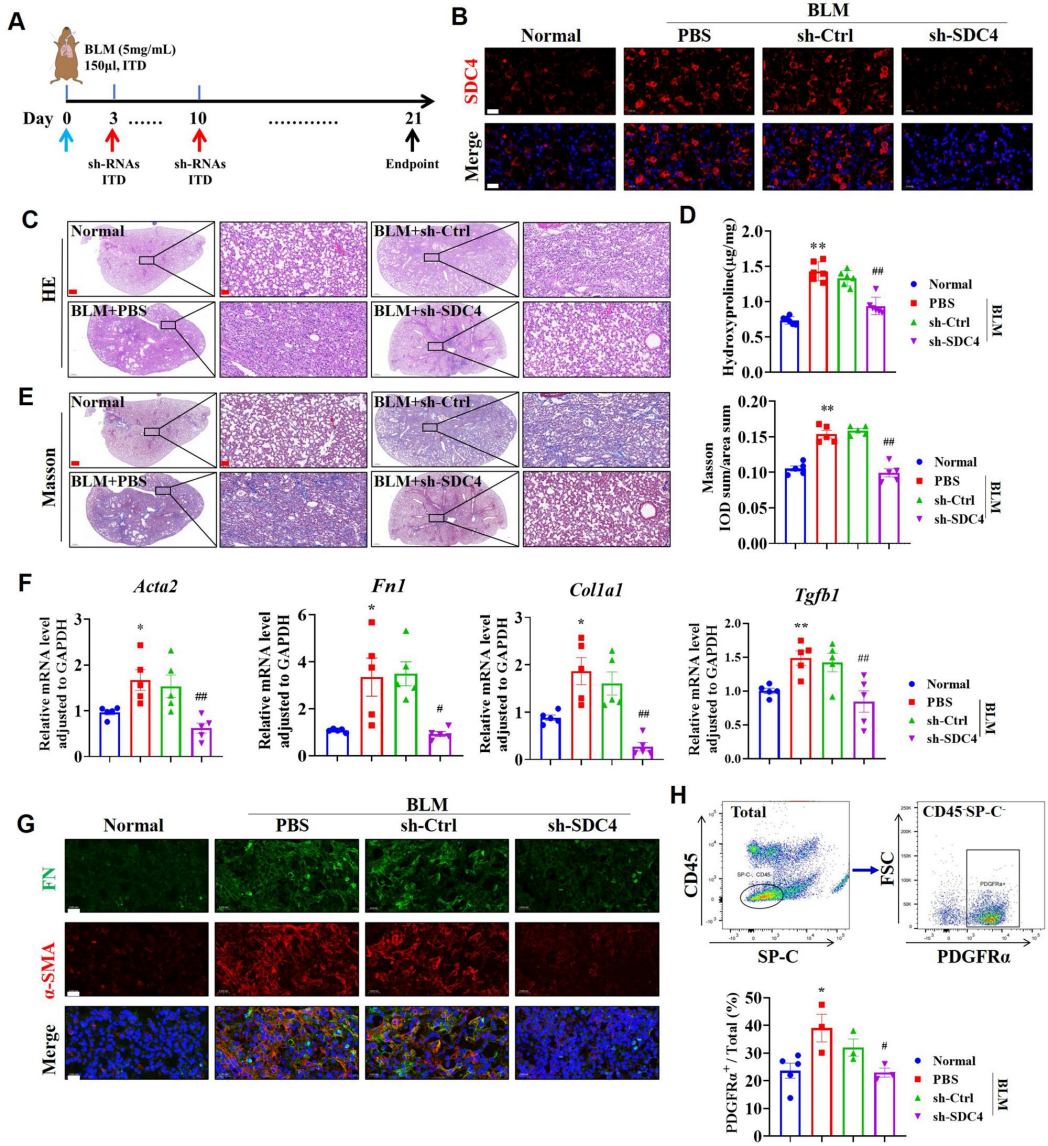
Knockdown of SDC4 alleviated PF and reduced fibroblast activation. (**A**) Flow chart of animal experiments. 1.5 × 10^7^ TU lentivirus carrying sh-Ctrl or sh-SDC4 was administrated via ITD on day 3 and day 10 post-BLM administration, and lung tissue samples were collected on day 21 (*n* = 6). (**B**) Immunofluorescence staining detected the level of SDC4 in lung tissue on day 21 (scale bar = 20 μm). (**C**) HE staining was performed (scale bars = 500 μm and 50 μm). (**D**) The content of collagen in lung tissue was quantified using a hydroxyproline assay (*n* = 6). (**E**) Masson staining and IOD analysis were conducted (scale bar= 500 μm and 50 μm, *n* = 5). (**F**) The mRNA levels of α-SMA, FN, Collagen I and TGF-β1 were measured by RT-PCR (*n* = 5). (**G**) The levels of α-SMA and FN were detected by immunofluorescence staining (scale bar= 20 μm). (**H**) Flow cytometry identified immune cells (CD45^+^), alveolar type II epithelial cells (SP-C^+^), and fibroblasts (PDGFRα^+^) in lung tissues (*n* = 3–5). Note: *P < 0.05, **P < 0.01 vs. normal group; #*P* < 0.05, ##*P* < 0.01 vs. BLM (PBS) group. ITD: oropharyngeal inhalation, PBS: Phosphate-Buffered Saline.

Oropharyngeal inhalation of lentivirus was used to therapeutically knock down SDC4 on days 3 and 10 following BLM inhalation (Figure 2A and B and Supplementary Figure S4A and B). BLM model group exhibited alveolar wall thickening and inflammatory cell infiltration, knockdown of SDC4 significantly reduced these lesions (Figure 2C). Measurements of hydroxyproline (Figure 2D), Masson staining and IOD value analysis (Figure 2E) indicated a significant increase in collagen deposition in the lung tissue of the BLM group, with smaller and uneven alveolar cavities and pathological remodeling of ECM. Knockdown of SDC4 significantly reduced collagen deposition, resulting in alveolar structure like that of the normal group (Figure 2C–E). Additionally, SDC4 knockdown significantly lowered the lung index in BLM mice (Supplementary Figure S4C) and reduced the numbers and proportions of inflammatory blood cells (Supplementary Figure S4D).

The mRNA levels of α-SMA, FN, Collagen I, TGF-β1 and the protein levels of α-SMA and FN were significantly increased in BLM lung tissue, and knockdown of SDC4 reversed these changes (Figure 2F and G). Flow cytometry revealed a higher ratio of CD45^-^SP-C^-^PDGFRα^+^ fibroblast in BLM lung tissue compared to the normal group, and SDC4 knockdown reduced the number of fibroblast (Figure 2H).

### dECM-PF promotes fibroblast activation by regulating integrin-αvβ1/FAK and TGF-β1/Smad2/3 pathways via SDC4, and affects inflammatory response by regulating PKCα

**Figure 3.**
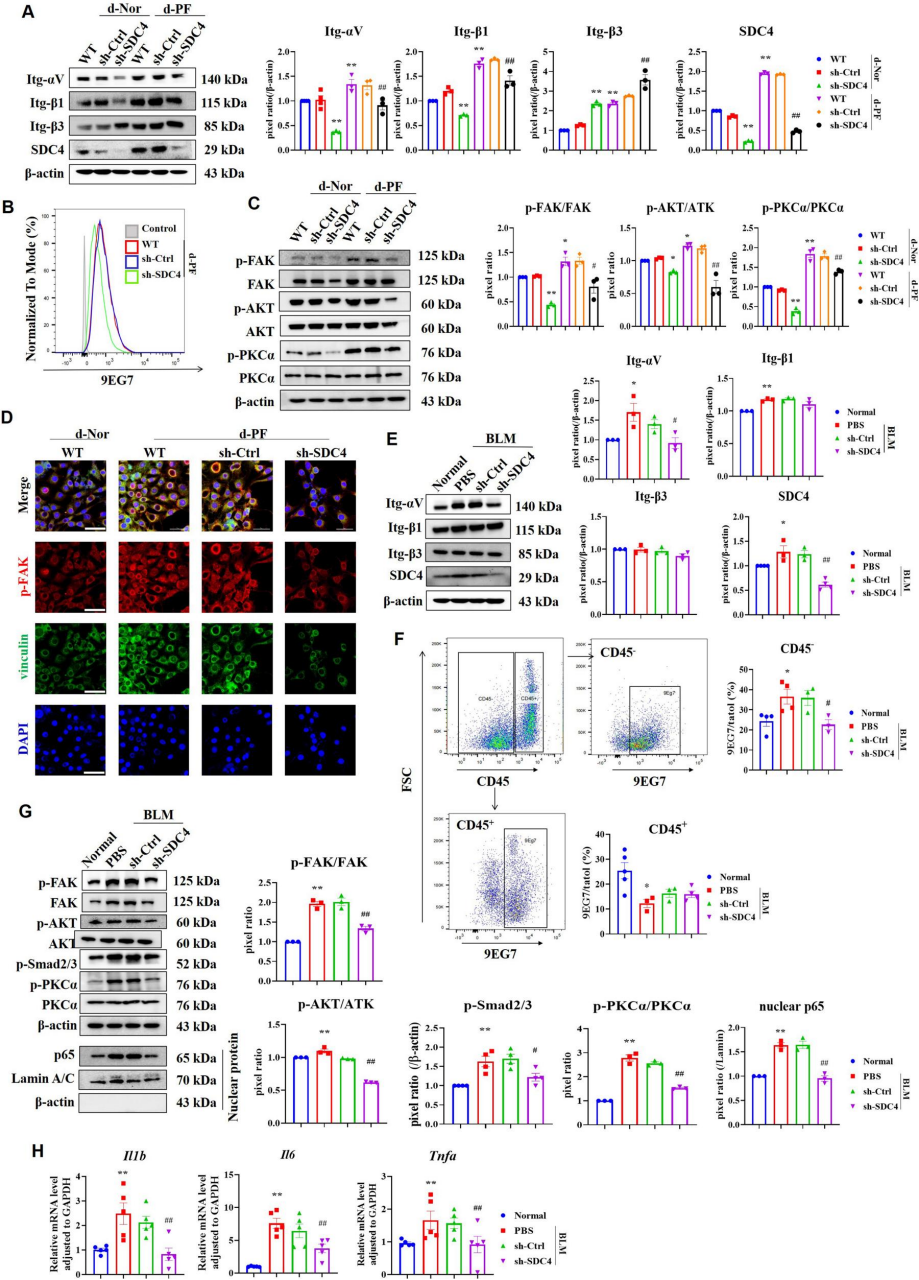
Knockdown of SDC4 inhibited integrin-αVβ1 and its downstream FAK/AKT pathway and TGF-β1/Smad2/3 pathway, and suppressed PKCα-inflammatory response. (**A**–**D**) NIH3T3 cells were seeded on d-Nor and d-PF, respectively, and cultured for 48 h. (**A**) The expression levels of integrin-αV, -β1, -β3, and SDC4 in SDC4 knocked-down fibroblast cells were detected, and the pixel ratio of each protein was analyzed using ImageJ (*n* = 3). (**B**) Flow cytometry detected the activation of integrin-β1 (9EG7) in fibroblast cells induced by d-PF. (**C**) The expression levels of indicated proteins were measured by Western blot (*n* = 3). (**D**) The formation of FA (co-localization of p-FAK and vinculin) in each group was observed by laser confocal microscopy (scale bar= 20 μm). Note: *P < 0.05, **P < 0.01 vs. WT in d-Nor group; #*P* < 0.05, ##*P* < 0.01 vs. WT in d-PF group. (**E**–**H**) Oropharyngeal inhalation of lentivirus knocked down the level of SDC4 in lung tissue and lung tissues were collected on the 21st day after BLM modeling (*n* = 6). (**E**) The expression levels of indicated proteins in mouse lung tissues were measured by Western blot (*n* = 3). (**F**) Flow cytometry was used to analyze the activation of integrin-β1 in lung tissues (*n* = 3–5). (**G**) The expression levels of indicated proteins in mouse lung tissues were detected and analyzed by ImageJ software (*n* = 3). (**H**) The mRNA levels of IL-6, IL-1β and TNF-α in lung tissues were analyzed by RT-PCR (*n* = 5). Note: *P < 0.05, **P < 0.01 vs. Normal group; #*P* < 0.05, ##*P* < 0.01 vs. PBS in BLM group. d-Nor: dECM-Nor, d-PF: dECM-PF.

In the culture of dECM+Fib, dECM-PF elevated the levels of SDC4, integrin-αv, -β1 and -β3. SDC4 knockdown resulted in the downregulation of integrin-αv and -β1, while integrin-β3 expression increased (Figure 3A), indicating a predominant effect on integrin-αvβ1 over integrin-αvβ3, which is consistent with the literature-reported specific association of SDC4 with the integrin-β1 subunit [34]. For integrin-β3 upregulation, we hypothesize that the compensatory upregulation of integrin-β3 may reflect a fibroblast-driven mechanism to maintain ECM adhesion: Integrin-β1 primarily binds fibronectin, whereas integrin-β3 interacts with vitronectin, both mediate cell-ECM adhesion [35, 36]. FN-coating experiments showed that SDC4 knockdown significantly inhibited the pan-activation of RGD-binding integrins (Supplementary Figure S5). Flow cytometry using the integrin-β1 activating antibody 9EG7 revealed that SDC4 knockdown decreased integrin-β1 activation (Figure 3B). Additionally, dECM-PF enhanced the levels of p-FAK and p-AKT, whereas SDC4 knockdown reduced the activation of FAK and AKT (Figure 3C), leading to diminished FA formation (Figure 3D). The FAK inhibitor PF-573228 significantly reduced dECM-PF-induced fibroblast activation and TGF-β1 upregulation in a dose-dependent manner (Supplementary Figure S6). These findings suggest that SDC4 plays a role in dECM-PF-induced fibroblast activation and TGF-β1 synthesis via the integrin-αvβ1/FAK pathway. Furthermore, SDC4 knockdown decreased the phosphorylation of its intracellular adapter protein, PKCα (Figure 3C).

*In vivo* results demonstrated increased expression levels of SDC4 and integrin-αv and -β1 in lung tissue, with no significant change in integrin-β3 (Figure 3E). SDC4 knockdown downregulated integrin-αv but did not notably affect β1 and β3 levels (Figure 3E). The activation level of integrin-β1 was significantly elevated in nonimmune cells (CD45^-^) in the BLM group, and SDC4 knockdown substantially reduced this activation. However, in immune cells (CD45^+^), integrin-β1 activation was significantly downregulated in the BLM group, and SDC4 knockdown had no significant effect (Figure 3F). The integrin-β1 subunit is the most abundant integrin, and our results show that although SDC4 has no significant effect on overall integrin-β1 expression in lung tissue, it notably reduces its activation in nonimmune cells. In PF tissue, p-FAK and p-AKT levels were elevated, and SDC4 knockdown lowered these levels (Figure 3G). Integrin-αvβ1 activates latent TGF-β1 *in vivo* [10]. Our results showed that p-Smad2/3 levels in fibrotic lung tissue were significantly increased, and SDC4 knockdown abolished this response (Figure 3G). Additionally, elevated p-PKCα levels were observed in fibrotic lung tissue, alongside increased nuclear NF-κB and inflammatory factors such as IL-1β, IL-6 and TNF-α (Figure 3G and H). SDC4 knockdown inhibited PKCα-NF-κB activation and downregulated IL-1β, IL-6 and TNF-α levels (Figure 3G and H). Treatment with PKCα inhibitor (PKCiota-IN-2 formic [37]) significantly inhibited dECM-PF-induced PKCα phosphorylation and NF-κB nuclear translocation (Supplementary Figure S7A), and downregulated the levels of inflammatory factors (Supplementary Figure S7B).

### SDC4-blocking antibody and SDC4_87-131_ peptide can abolish dECM-PF-induced extracellular interaction between SDC4 and integrin-αvβ1

**Figure 4.**
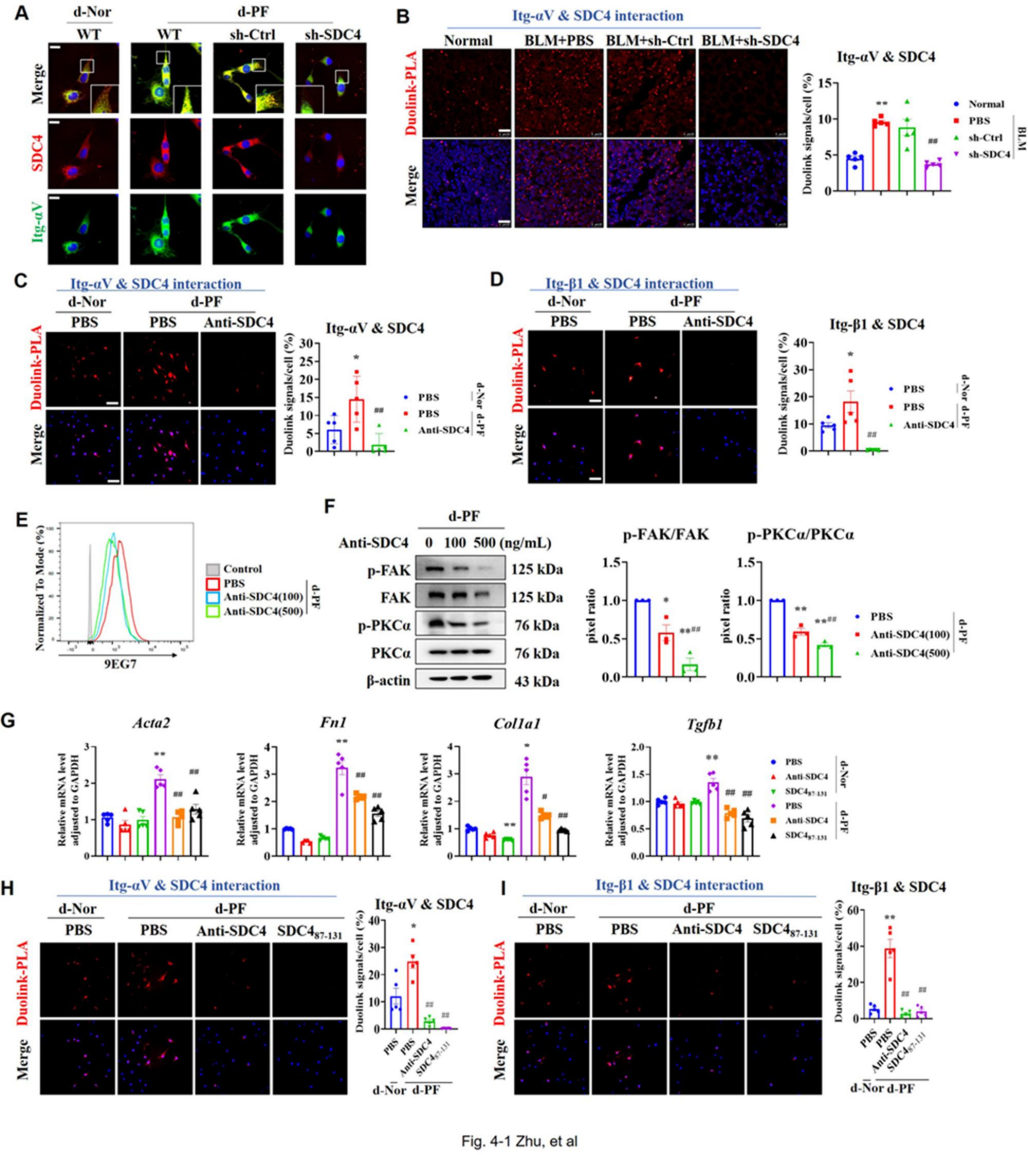
A significant extracellular domain interaction between SDC4 and integrin-αVβ1 was observed, and both the blocking antibody Anti-SDC4_(93-121)_ and the interfering peptide SDC4_87-131_ abolished this extracellular interaction. (**A**) Colocalization of SDC4 and integrin-αV was detected by laser confocal microscopy (scale bar = 5 μm). (**B**) Extracellular interaction between integrin-αV and SDC4 in lung tissues 21 days after BLM administration was detected by Duolink-PLA method (scale bar = 25 μm) and analyzed by ImageJ (*n* = 5). Note: **P < 0.01 vs. Normal group; ##*P* < 0.01 vs. PBS in BLM group. (**C**, **D**) The effects of Anti-SDC4_(93-121)_ (500 ng/mL) on the extracellular interaction of SDC4/integrin-αV (**C**) and SDC4/integrin-β1 (**D**) were detected by Duolink-PLA (scale bar= 20 μm, *n* = 5). Note: *P < 0.05 vs. PBS in d-Nor group; ##*P* < 0.01 vs. PBS in d-PF group. (**E**) Flow cytometry analysis showed that Anti-SDC4_(93-121)_ (100, 500 ng/mL) could inhibit the activation of integrin-β1 in fibroblast cells. (**F**) Western blot analysis showed that Anti-SDC4_(93-121)_ (100, 500 ng/mL) could inhibit the activation of FAK and PKCα (*n* = 3). Note: *P < 0.05, **P < 0.01 vs. PBS group; ##*P* < 0.01 vs. Anti-SDC4(100) group. (**G**) The effects of 100 nM SDC4_87-131_ and 100 ng/mL Anti-SDC4_(93-121)_ on α-SMA, FN, Col I and TGF-β1 were examined (*n* = 5). Note: *P < 0.05, **P < 0.01 vs. PBS in d-Nor group; #*P* < 0.05, ##*P* < 0.01 vs. PBS in d-PF group. (H) and (I) The effects of SDC4_87-131_ (100 nM) on the extracellular interaction of SDC4/integrin-αV (**H**) and SDC4/integrin-β1 (**I**) were detected by Duolink-PLA (scale bar= 20 μm, *n* = 5). Note: *P < 0.05, **P < 0.01 vs. PBS in d-Nor group; ##*P* < 0.01 vs. PBS in d-PF group. d-Nor: dECM-Nor, d-PF: dECM-PF.

Immunofluorescence staining revealed that dECM-PF enhanced the colocalization of SDC4 with integrin-αv (Figure 4A). Duolink-PLA method observed increased extracellular interactions between SDC4 and integrin-αv in BLM lung tissue, and knockdown of SDC4 significantly reduced the fluorescence produced by proximity ligation (Figure 4B).

It has been reported that polyclonal antibodies prepared using the extracellular segment of SDC4 (N93-V121) as the antigen achieved the same relief as knocking down SDC4 in experimental asthma and rheumatoid arthritis [21, 22]. We prepared Anti-SDC4_(93-121)_ polyclonal antibodies (Supplementary Figure S8A), and found that Anti-SDC4_(93-121)_ (500 ng/mL) reduced the activation of pan-integrins (Supplementary Figure S8B). Duolink-PLA assay indicated that dECM-PF significantly upregulated the proximity ligation of integrin-αv, integrin-β1 with SDC4, respectively, and Anti-SDC4_(93-121)_ could almost completely inhibit these interactions (Figure 4C and D). Simultaneously, Anti-SDC4_(93-121)_ reduced integrin-β1 activation (Figure 4E), and downregulated the phosphorylation of FAK and PKCα in a dose-dependent manner (Figure 4F). Researches have demonstrated that SDC4's regulation of integrin-β1 activation is contingent upon their co-localization [34], and that tension applied to SDC4 induces integrin-β1 activation [38]. Our results showed that the Anti-SDC4_(93-121)_ antibody blocked the extracellular interaction of SDC4 and integrin-αvβ1 induced by dECM-PF, thereby inhibiting the activation of integrin-αvβ1/FAK pathway and SDC4/PKCα pathway.

SDC4_87-131_ is a synthetic peptide of the N87-M131 segment of the extracellular domain of SDC4 (Supplementary Figure S9A). Study has reported that SDC4_87-131_ can inhibit cell migration comparably to the exogenous full-length SDC4 [39]. In the concentration range of 10–1000 nM, SDC4_87-131_ exhibited no significant toxicity to fibroblast (Supplementary Figure S9B). It was determined that 100 nM SDC4_87-131_ could significantly inhibit dECM-PF-induced fibroblast activation (Supplementary Figure S9C), thus, establishing the dosage concentration of SDC4_87-131_ at 100 nM. Comparing the effects of SDC4_87-131_ (100 nM) and Anti-SDC4_(93-121)_ (100 ng/mL), it was found that SDC4_87-131_ inhibited dECM-PF-induced fibroblast activation and TGF-β1 synthesis, and its effect is similar to that of Anti-SDC4_(93-121)_ (Figure 4G). Duolink-PLA assay found that SDC4_87-131_ and Anti-SDC4_(93-121)_ could significantly reduce the integrin-αv/SDC4 and integrin-β1/SDC4 interactions induced by dECM-PF (Figure 4H and I).

### SDC4_87-131_ exhibited anti-PF effects and displayed a more significant anti-inflammatory effect than integrin-αvβ1 inhibitor

**Figure 5.**
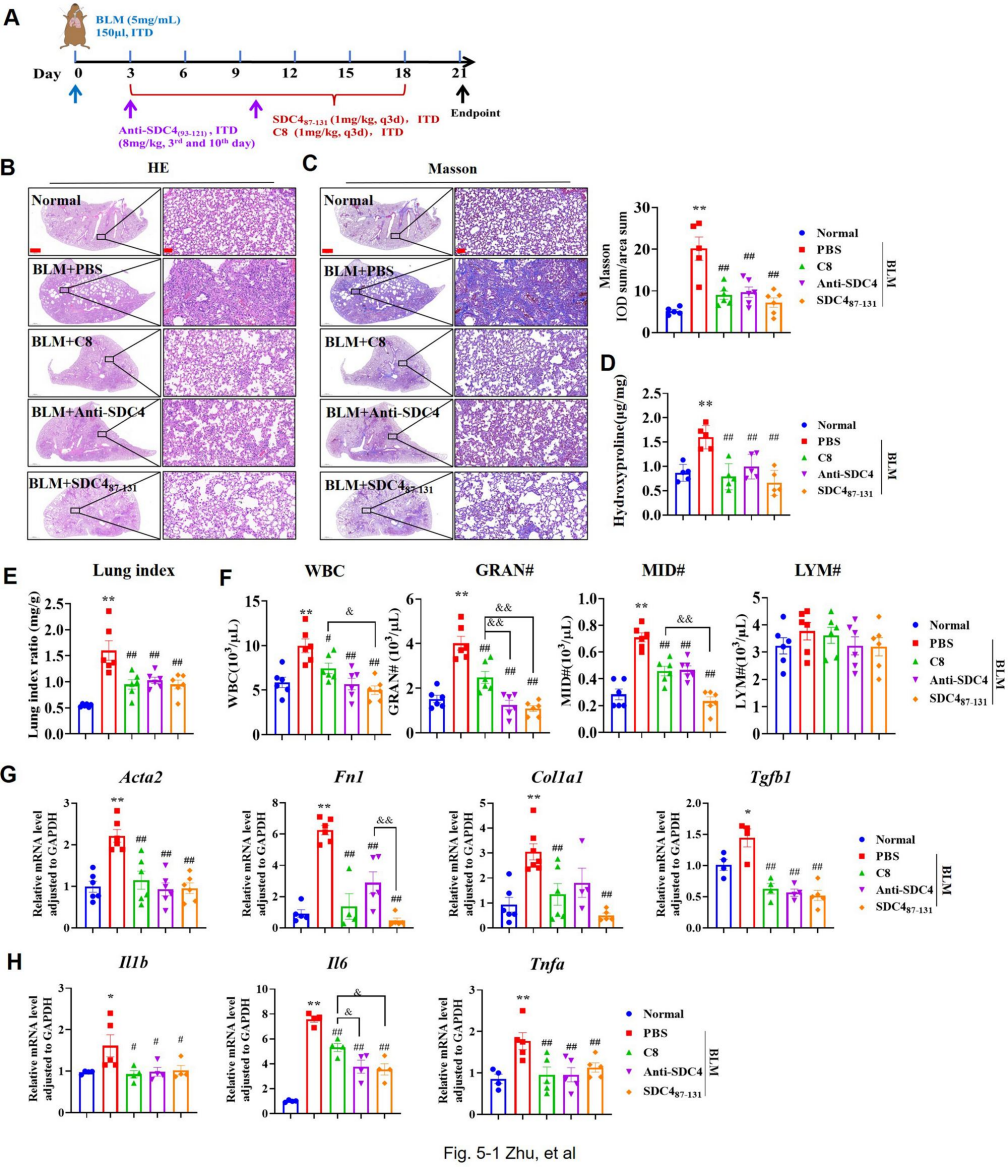
SDC4_87-131_ alleviated BLM-induced PF and reduced the inflammatory response of lung tissue. (**A**) Flowchart of animal experiments. Drug administration commenced on day 3 after oropharyngeal instillation of BLM, and the experiment ended at 21th day (*n* = 6). (**B**, **C**) HE staining (**B**), Masson staining and IOD analysis of collagen deposition (**C**) in the SDC4_87-131_, C8 and Anti-SDC4_(93-121)_ treatment group (scale bar = 1000 μm, 50 μm). (**D**) The collagen content in lung tissue was measured by hydroxyproline assay (*n* = 5). (**E**) Pulmonary index (*n* = 6). (**F**) Blood leukocyte count (*n* = 6). (**G**, **H**) RT-PCR analysis of α-SMA, FN, Col I and TGF-β1 (**G**) (*n* = 5–6), and IL-1β, IL-6 and TNF-α (**H**) (*n* = 4–5). Note: *P < 0.05, **P < 0.01 vs. Normal group; #*P* < 0.05, ##*P*< 0.01 vs. PBS in BLM group. ITD: oropharyngeal inhalation, WBC: white blood cell, GRAN: neutrophil, MID: middle cell, LYM: lymphocyte.

Based on the dosage of SDC2 fragment peptide [40] and our experimental results (Supplementary Figure S10A and B), the dosage schedule of SDC4_87-131_ was set as follows: starting on the third day after BLM inhalation, 1 mg/kg, administered via trachea infusion once every three days. And Anti-SDC4_(93-121)_ 8 mg/kg was administered on the third and tenth days (reference dosage [41]). Integrin-αvβ1 inhibitor compound C8 has been reported to have anti-PF activity [35] and was used as a control drug in this experiment (Figure 5A).

Results from HE staining (Figure 5B), Masson staining and IOD analysis (Figure 5C) and hydroxyproline determination (Figure 5D) indicated that each treatment group could alleviate alveolar structural disorders and collagen deposition, with no significant difference in the effects of SDC4_87-131_, Anti-SDC4_(93-121)_ and C8 (Figure 5B–D). Moreover, each treatment group could reduce the lung index and blood leukocyte count in BLM mice (Figure 5E and F). Among these, the downregulating effect of SDC4_87-131_ was notably stronger than that of C8 (Figure 5F). Additionally, SDC4_87-131_ significantly reduced the mRNA levels of α-SMA, FN, Col I, TGF-β1, IL-1β, IL-6 and TNF-α in the lung tissue of BLM mice, with SDC4_87-131_ and Anti-SDC4_(93-121)_ showing a stronger effect in downregulating IL-6 than C8 (Figure 5G and H).

### SDC4_87-131_ inhibits the integrin-αvβ1/FAK, TGF-β1/Smad2/3 pathways and PKCα-inflammatory response by interfering with the fibrotic ECM-induced interaction between SDC4 and integrin-αvβ1

**Figure 6.**
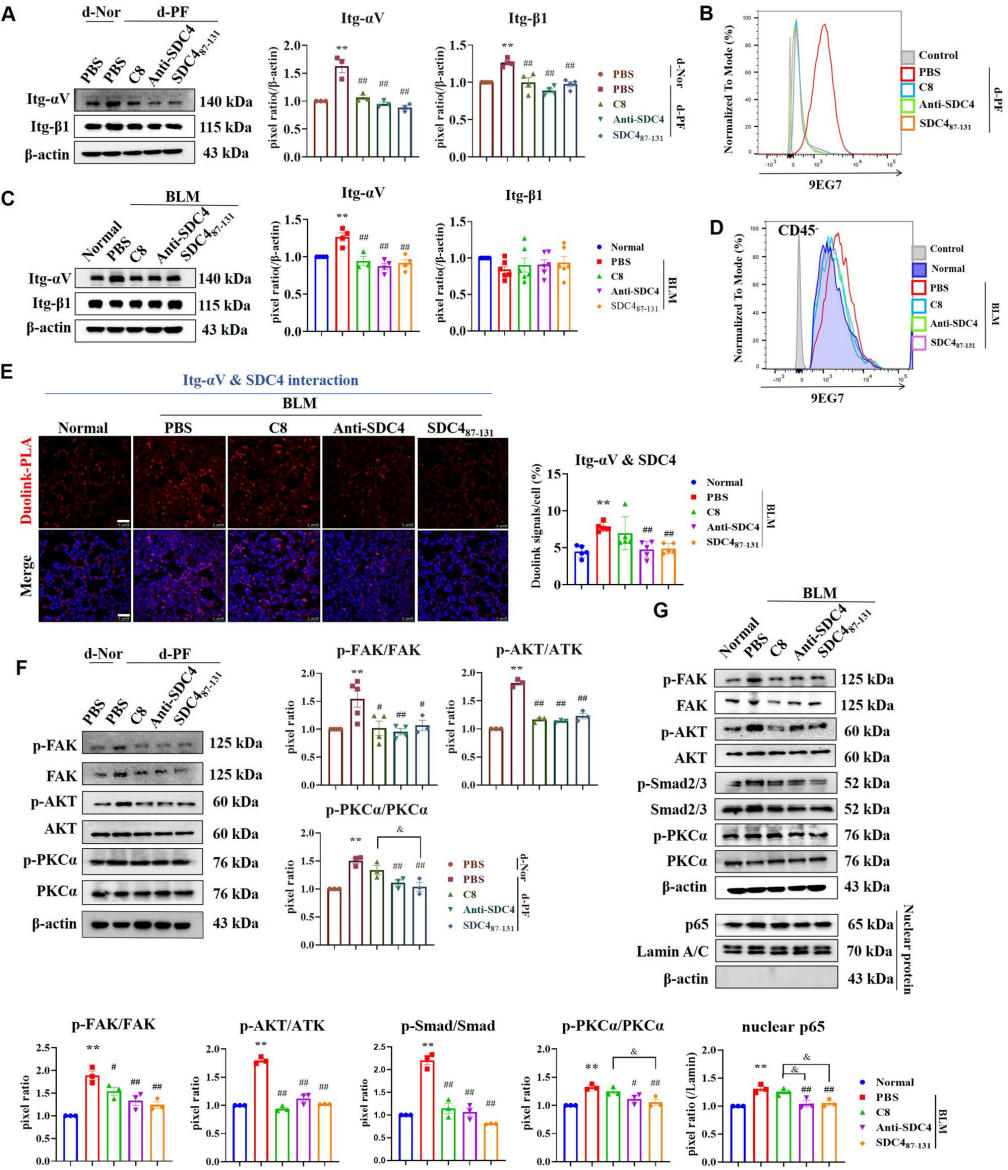
SDC4_87-131_ reduced the activation of integrin-αVβ1 and inhibited the activity of related signaling pathways. (**A**, **B**) Fibroblast was cultured on d-Nor and d-PF, and treated with SDC4_87-131_ (100 nM), C8 (20 nM) and Anti-SDC4_(93-121)_ (100 ng/mL) for 48 hr. (**A**) The expression level of integrin-αVβ1 was detected by Western blot (*n* = 3). Note: **P < 0.01 vs. PBS in d-Nor group; ##*P* < 0.01 vs. PBS in d-PF group. (**B**) The activation level of integrin-β1 was detected by Flow cytometry. (**C**–**E**) The expression level of integrin-αVβ1 in BLM lung tissue was detected by Western blot (C, *n* = 3), and the activation level of integrin-β1 was detected by Flow cytometry (**D**). (**E**) The impact of SDC4_87-131_, C8 and Anti-SDC4_(93-121)_ on the interaction between integrin-αV and SDC4 was observed using the Duolink-PLA method (scale bar= 25 μm, *n* = 5). Note: SDC4_87-131_: 1 mg/kg, ITD, q3d; C8: 10 mg/kg, IP, qd; Anti-SDC4_(93-121)_ 8 mg/kg, ITD, the third and 10th day after BLM administration and the experiment was concluded on the 21st day. **P < 0.01 vs. Normal group; ##*P* < 0.01 vs. PBS in BLM group. (**F**) The expression levels of indicated proteins were detected by Western blot, and the pixel ratios were analyzed using ImageJ (*n* = 3). Note: **P < 0.01 vs. PBS in d-Nor group; #*P* < 0.05, ##*P* < 0.01 vs. PBS in d-PF group; &&*P* < 0.01. (**G**) The expression levels of indicated proteins in mouse lung tissues were detected by Western blot (*n* = 3). Note: *P < 0.05, **P < 0.01 vs. Normal group; ##*P*< 0.01 vs. PBS in BLM group; &&*P* < 0.01. d-Nor: dECM-Nor, d-PF: dECM-PF.

SDC4_87-131_ could inhibit pan-integrins activation (Supplementary Figure S11). In both *in vitro* and *in vivo* PF models, SDC4_87-131_ significantly reduced the expression level of integrin-αv, while the expression of integrin-β1 was only reduced in the *in vitro* PF model (Figure 6A and C). SDC4_87-131_ significantly inhibited dECM-PF-induced integrin-β1 activation, as did C8 (Figure 6B). In mouse BLM lung tissue, upregulated integrin-β1 activation in the nonimmune cell population (CD45^-^) was abolished by SDC4_87-131_, equivalent to the effects of C8 and Anti-SDC4_(93-121)_ (Figure 6D). Duolink-PLA assay results demonstrated that SDC4_87-131_ and Anti-SDC4_(93-121)_ could reduce the interaction of integrin-αv and SDC4, but C8 had no such effect (Figure 6E). And SDC4_87-131_ was found to inhibit FAK/AKT phosphorylation activation in both *in vitro* and *in vivo* experiments (Figure 6F and G). In addition, SDC4_87-131_ reduced p-Smad2/3 protein levels in BLM lung tissue (Figure 6G), and its effect was not significantly different from C8 and Anti-SDC4. The difference was that SDC4_87-131_ and Anti-SDC4_(93-121)_ could downregulate the phosphorylation level of PKCα and the nuclear content of NF-κB (p65), while C8 has no significant effect on these (Figure 6F and G). These results indicate that, compared with integrin-αvβ1 inhibitor, SDC4_87-131_ and Anti-SDC4_(93-121)_ have the advantage of inhibiting both the functions of integrin-αvβ1 and SDC4.

### Optimized design of SDC4_87-131_

**Figure 7.**
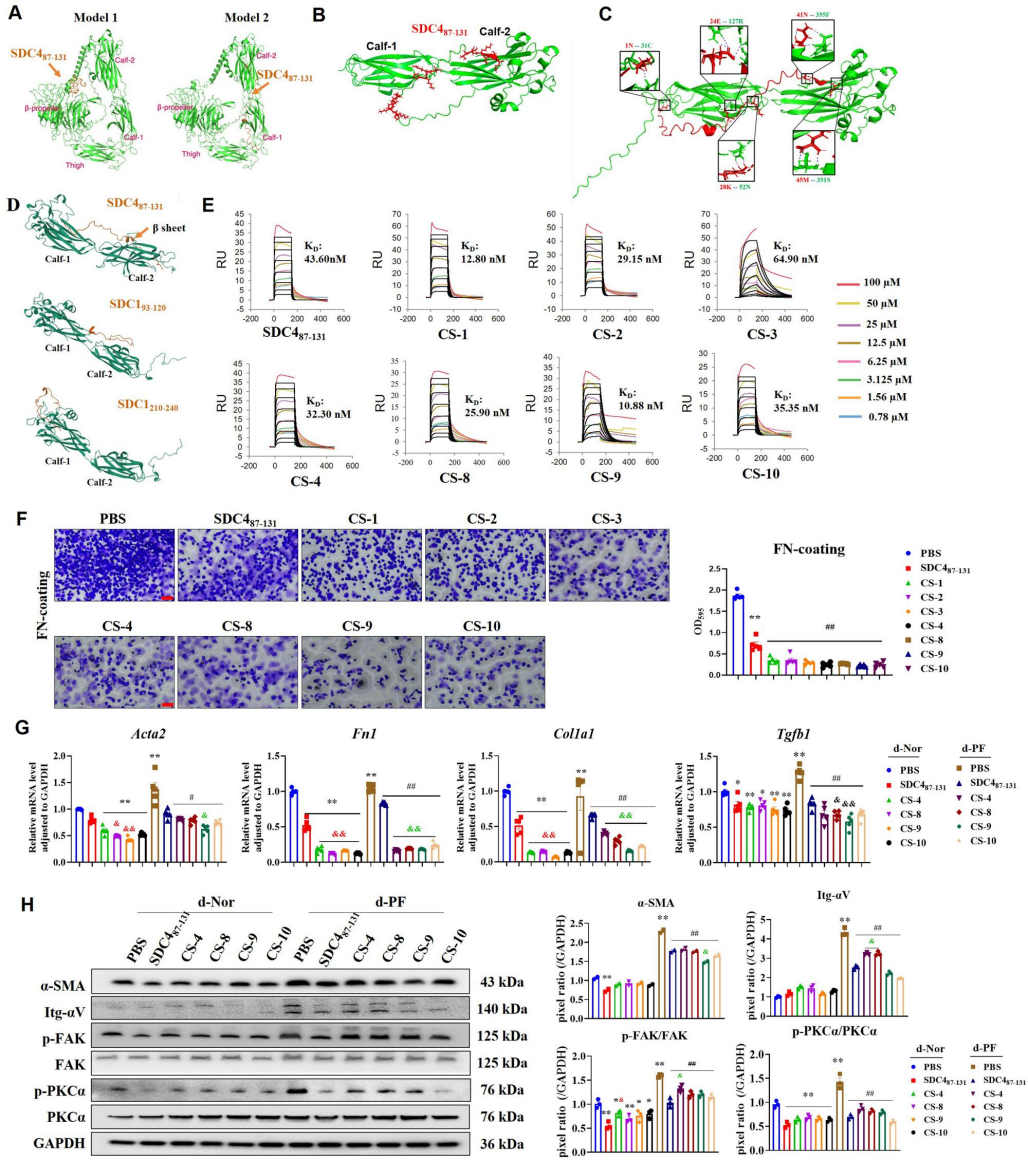
Predicted binding mode of SDC4_87-131_ to integrin-αV and SPR determination of the affinity, and *in vitro* anti-PF activity of designed peptides. (**A**–**D**) The binding mode of SDC4_87-131_ to integrin-αV was predicted by using the AlphaFold2-Multimer model. (**A**) Two major binding modes of SDC4_87-131_ to the entire extracellular domain of the integrin-αV. (**B**) Predictive binding pattern of SDC4_87-131_ to the Calf-1/Calf-2 domain of integrin-αV. (**C**) Molecular docking sites. (**D**) Predicted binding patterns of three polypeptides with the Calf-1/Calf-2 domain. (**E**) SPR was used to detect the binding of peptides to integrin-αVβ1, and the affinity value *K*_*d*_ was calculated. (**F**) FN-coating cell adhesion assay was employed to indirectly assess the activation level of pan-integrins (the concentration of all peptides was 100 nM, scale bar= 25 μm, *n* = 4–5). Note: **P < 0.01 vs. PBS; #*P* < 0.05, ##*P* < 0.01 vs. SDC4_87-131_. (**G**) The mRNA levels of α-SMA, FN, Col I and TGF-β1 were detected by RT-PCR (*n* = 5). (**H**) The expression levels of indicated proteins were detected by Western blot (*n* = 3). Note: *P < 0.05, **P < 0.01 vs. PBS in d-Nor group; #*P* < 0.05, ##*P* < 0.01 vs. PBS in d-PF group; &*P* < 0.05, &&*P* < 0.01 vs. SDC4_87-131_ group. d-Nor: dECM-Nor, d-PF: dECM-PF.

The AlphaFold2^®^-Multimer [28] model was employed to simulate the binding of the SDC4_87-131_ peptide to the entire extracellular domain of the integrin-αv, yielding two main binding modes (Figure 7A). The binding free energy of SDC4_87-131_ with the Calf-1/Calf-2 domain of αV (Model 2) was lower than that with the β-propeller domain of αV (Model 1 and Supplementary Table S1). Subsequently, a binding model of the SDC4_87-131_ and the αV Calf-1/Calf-2 domain was constructed (Figure 7B), and it showed that the C-terminus, middle part and N-terminus of SDC4_87-131_ had binding sites with a confidence level exceeding 0.8 (Supplementary Figure S12 and Figure 7C).

Two active peptide fragments from SDC1 [42]: SDC1_93-120_ and SDC1_210-240_, were used. Upon comparing the binding modes of the three peptides, SDC4_87-131_ had more contact areas with Calf-1/Calf-2, and the C-terminus of SDC_87-131_ could form a stable β-sheet (Figure 7D). At the same time, the Models’ similarity of SDC4_87-131_ was the highest among the three peptides, and the binding free energy was the lowest (Supplementary Table S2), indicating that the binding of SDC4_87-131_ to integrin-αv exhibits certain selectivity.

Subsequently, based on SDC4_87-131_, we designed more than 50 peptide sequences using the Protein MPNN algorithm and structure-based template search, and simulated the binding modes of these peptides with integrin-αv Calf-1/Calf-2, of which 30 predicted template modeling scores were similar to those of SDC4_87-131_ (Supplementary Table S3), and 10 peptide sequences with a length of ≤40 aa and binding free energy similar to or lower than that of SDC4_87-131_ were screened out (Supplementary Tables S4 and S5). Among them, CS-5, CS-6 and CS-7 could not be successfully synthesized (see Supplementary Figure S13 for HPLC and MS spectra of the peptides).

Surface plasmon resonance (SPR) results showed that the affinity of SDC4_87-131_ and 7 designed peptides to integrin-αvβ1 was at nM level (Figure 7E). Among them, the KD values of several designed peptides were lower than those of SDC4_87-131_, indicating that virtual screening and SPR detection have certain repeatability.

### Designed peptides suppress dECM-PF-induced fibroblast activation more effectively than SDC4_87-131_

The designed peptides exhibited no significant toxicity to fibroblast within the range of 10–10 000 nM (Supplementary Figure S14). The inhibitory effect of CS-3, CS-4, CS-8, CS-9 and CS-10 on pan-integrins activation was stronger than that of SDC4_87-131_ (Figure 7F). These polypeptides could significantly downregulate the mRNA levels of α-SMA, FN, Col I and TGF-β1 induced by dECM-Nor and dECM-PF, and their effects on FN and Col I were significantly greater than those of SDC4_87-131_ (Figure 7G), and the downregulation effect of CS-9 on all four molecules was significantly higher than that of SDC4_87-131_. They could significantly decrease the protein levels of α-SMA, integrin-αv, p-FAK and p-PKCα, where CS-4 exhibited a weaker reduction in integrin-αv and p-FAK compared to SDC4_87-131_, while CS-9 exhibited a stronger reduction in α-SMA than SDC4_87-131_ (Figure 7H).

### The anti-PF effect of the designed peptide CS-9 is superior to that of SDC4_87-131_

**Figure 8.**
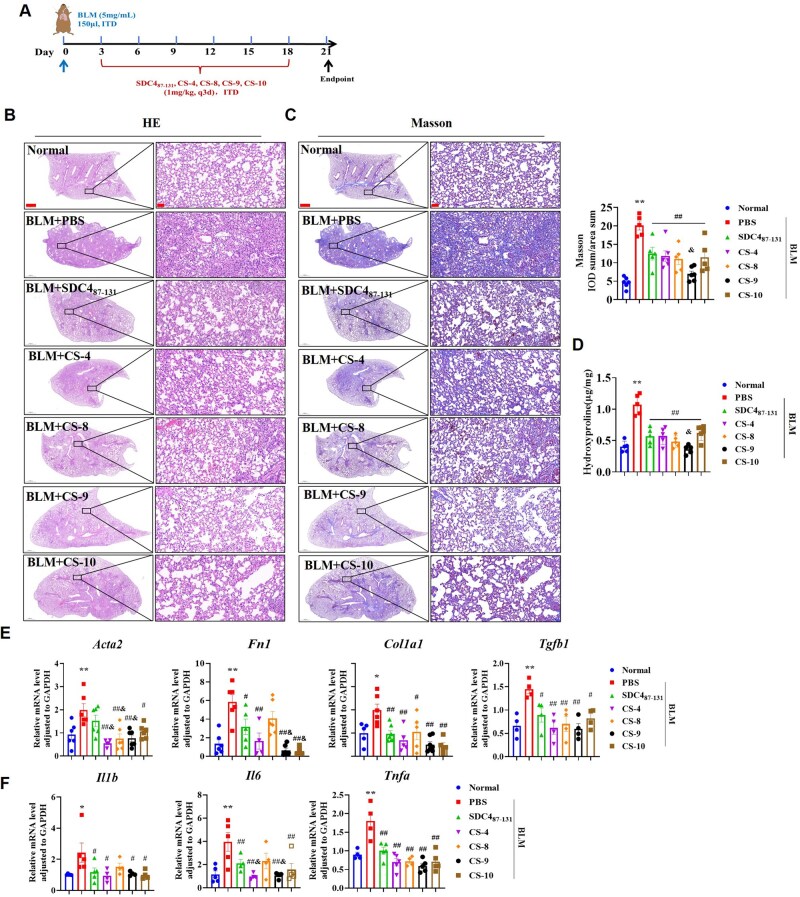
Designed peptides exhibited the ability to alleviate BLM-induced PF. (**A**) Flow chart of animal experiments. Dosing began on day 3 after oropharyngeal inhalation of BLM, and the experiment ended at 21th days (*n* = 6). (**B**, **C**) HE staining (**B**), Masson staining and IOD analysis of collagen deposition (**C**) in each group (scale bar =1000 μm, 50 μm). (**D**) The content of collagen in lung tissue was measured by hydroxyproline assay (*n* = 5). (**E**, **F**) RT-PCR assessment of α-SMA, FN, Col I and TGF-β1 (**E**), and IL-1β, IL-6 and TNF-α (**F**) (*n* = 4–6). Note: *P < 0.05, **P < 0.01 vs. Normal group; #*P* < 0.05, ##*P* < 0.01 vs. PBS in BLM group; &*P* < 0.05, &&*P* < 0.01 vs. SDC4_87-131_ group. ITD: oropharyngeal inhalation, FN: fibronectin, Col: collagen I.

The designed peptides were administered on the third day after BLM modeling, at 1 mg/kg by tracheal instillation, once every three days (Figure 8A). Results showed that each treatment group reduced the structural disorder and remodeling of lung tissue in BLM mice and decreased collagen deposition, with CS-9 exhibiting a significantly stronger inhibitory effect on collagen deposition than SDC4_87-131_ (Figure 8A–D). Each treatment group reduced the lung index and the count or proportion of neutrophils and middle cells (Supplementary Figure S15A and B). The number of lymphocytes, reduced in BLM-induced PF, was increased by the designed peptide (Figure S15B). The mRNA levels of α-SMA, FN, Col I and TGF-β1 were reduced in all peptide treatment group (Figure 8E). Simultaneously, levels of inflammatory factors in lung tissue were downregulated (Figure 8F). Among these, the effects of CS-9 on α-SMA, FN and IL-6 were significantly stronger than that of SDC4_87-131_. Taking together, CS-9 showed significantly enhanced anti-PF effect compared with SDC4_87-131_. We observed the dynamic distribution of CS-9 in lung tissue and found that fluorescently labeled CS-9 reached peak fluorescence intensity and spread throughout the lung tissue 0.5 hr after intratracheal administration, and then, gradually decreased within the next 6 hr (Supplementary Figure S16). The distribution characteristics of CS-9 in the lung further support its potential application in PF.

## Discussion

This study identified for the first time that fibrotic lung ECM promotes fibroblast activation through SDC4/integrin-αvβ1 interaction, driving the continuous progression of PF. Our results showed that SDC4, integrin-αv and -β1 are persistently highly expressed in PF and knocking down SDC4 in lung tissue can alleviate PF lesions. The literature reports that the SDC4 promoter region contains a Smad3 binding sequence, and the TGF-β/Smad3 signaling pathway can enhance SDC4 transcription [43]. Thus, the widespread presence of TGF-β in PF supports the high expression of SDC4. In the early stages of IPF, patients with high SDC4 levels had significantly worse prognosis than those with low levels [44]. Jiang *et al*. reported increased SDC4 expression in BLM-induced PF [45]. Our study further observed high levels of SDC4, integrin-αv and -β1 from days 7–17 after BLM stimulation. Simultaneously, therapeutic knockdown of SDC4 in lung tissue could significantly alleviate PF, accompanied by a significant reduction in inflammatory cells and factors, supporting the targeting of SDC4 for therapeutic intervention in PF.

The role of SDC4 in ECM-promoted fibroblast activation has not been studied. This study found that SDC4 knockdown reduced dECM-PF-induced fibroblast activation. In BLM-induced PF, SDC4 knockdown alleviated ECM pathological remodeling and fibroblast activation, indicating a weakened positive feedback loop between fibroblast activation and ECM remodeling. Our results suggested that fibrotic ECM mediates fibroblast activation and PF progression via SDC4/integrin-αvβ1 interaction and the associated FAK/AKT, TGFβ1/Smad2/3 and PKCα/NF-κB pathways. During healing, integrin expression is often regulated by ECM exposure and growth factors [46]. Our study found that SDC4 influences integrin-αvβ1 expression and activation, its knockdown significantly reduces integrin-β1 activation in fibroblast. Researches have reported that SDC4 and integrin together mediate the conversion of ECM mechanical signals into cellular biochemical signals, such as FAK [14, 17]. This study demonstrated that knocking down SDC4 inhibited the phosphorylation activation of FAK/AKT downstream of integrin-αvβ1. Both other research [47] and our experiments have confirmed that FAK activity is closely related to fibroblast activation. Furthermore, integrin-αvβ1 has been reported to be involved in PF by regulating TGF-β1 activation, and inhibition of integrin-αvβ1 can alleviate BLM-induced PF in mice [35, 48]. We investigated the TGF-β1/p-Smad2/3 pathway in lung tissue and found that knocking down SDC4 blocked the synthesis of TGF-β1 by fibroblast and reduced the phosphorylation of Smad2/3. Our experimental results demonstrated that SDC4 not only regulated the activation of integrin-αvβ1, but also modulated its expression (Figure 3A). Studies have reported that knockdown of SDC4 in endothelial cells downregulated the expression of integrin-α5β1 and -αvβ3 [49]. Regarding its mechanism of action, SDC4 enhances PKCα activation (Figure 3C), and PKCα is reported to be a key regulator of integrin function [36]. Shuang Cai et al. found that NF-κB increases integrin-αvβ3 expression [50], while FoxO3a, another PKCα-regulated transcription factor, modulates integrin-α5 expression [51, 52]. Collectively, these findings suggest that SDC4 knockdown attenuates PKCα activation, potentially suppressing integrin-αvβ1 expression via reduced NF-κB/FoxO3a activity.

This study also found that SDC4 knockdown could significantly reduce the inflammatory response in PF. Fibrosis is characterized as a chronic inflammatory process, and inhibiting inflammation is crucial in managing PF [53, 54]. The regulatory effect of SDC4 on inflammation cannot be fully explained by its impact on integrin-αvβ1 and related signaling pathways. Studies have shown that ECM biomechanical signals can cause SDC4 to recruit and activate PKCα in the intracellular domain [38]. PKCα activation enhances multiple signaling pathways, such as ERK1/2 and NF-κB [17, 19]. Knocking down SDC4 has been shown to diminish the inflammatory response in allergic asthma and rheumatoid arthritis [19, 22, 55]. This study revealed that the stimulatory effect of fibrotic lung ECM on SDC4 also aggravated tissue inflammation. Knocking down SDC4 can downregulate the phosphorylation activation of PKCα, reduced the nuclear transfer of NF-κB (p65), lowered the levels of inflammatory factors IL-1β, IL-6 and TNF-α, and decreased the number of blood inflammatory cells, demonstrating a significant anti-inflammatory effect.

Researches have reported that SDC4 regulates the activation of integrin-β1 through co-localization with it [34]. In this study, the SDC4 blocking antibody, Anti-SDC4_(93-121)_, eliminate the proximal interaction between SDC4 and integrin-αvβ1 induced by dECM. Meanwhile, integrin-β1 activation, FN protein adhesion and FAK activation were inhibited. This confirmed that the interaction between SDC4 and integrin-αvβ1 in the vicinity of SDC4 (N93-V121) is a prerequisite for the execution of the above functions, laying the foundation for the application of interfering peptides.

Syndecans and integrins collaboratively regulate FA formation and cell adhesion [17, 56]. PKCα is known to modulate integrin-mediated FAK phosphorylation and FA assembly [56, 57]. Inhibition of PKCα not only blocks NF-κB activation but also reduces FAK phosphorylation [58]. Conversely, FAK activation can induce NF-κB activity [59], highlighting a bidirectional crosstalk between the SDC4/PKCα/NF-κB and integrin/FAK pathways. It has been reported that using SDC4_87-131_ reduces cell adhesion and migration [39]. Our study first demonstrated the anti-PF activity of SDC4_87-131_ both *in vivo* and *in vitro*. This peptide disrupts the SDC4/integrin-αvβ1 interaction, thereby inhibiting the functions of dECM-PF-associated integrin-αvβ1/FAK and SDC4/PKCα/NF-κB. Compared with the integrin-αvβ1 inhibitor Compound C8, SDC4_87-131_ not only has a similar effect of inhibiting FAK and Smad2/3 activation but also has a stronger anti-inflammatory effect by inhibiting the PKCα/NF-κB pathway. Based on the anti-PF activity of SDC4_87-131_, a series of peptides were designed aiming to enhance anti-PF activity. The designed peptides were screened by calculating the binding free energy, and CS-1∼CS-4, CS-8∼CS-10 were obtained, which were less than 35 aa in length and had a maximum of about 40% new sequences compared with SDC4_87-131_. *In vitro* and *in vivo* activity evaluation showed that compared with SDC4_87-131_, CS-9 significantly enhanced anti-integrin-αvβ1 activation and Anti-SDC4 effect, reduced fibroblast activation and showed optimized effects in inhibiting PF. CS-9’s low toxicity, defined mechanism and inhalable delivery route position it as a promising clinical candidate. Future studies should compare its efficacy with pirfenidone and nintedanib in advanced PF models.

## Conclusion

This study revealed the interaction between fibrotic lung ECM and fibroblast, the key functional cell of fibrosis and developed a novel peptide to hinder this interaction, thereby exhibiting significant anti-PF activity. This study may provide new ideas for the research of biopharmaceuticals against PF. However, the universality of CS-9 in different pulmonary fibrosis models and the design of pulmonary drug delivery carriers need further study.

## Supplementary Material

rbaf057_Supplementary_Data

## Data Availability

The mass spectrometry proteomics data of fibroblast induced by dECM have been deposited to the ProteomeXchange Consortium with the dataset identifier PXD042526 (https://www.iprox.cn/page/SSV024.html; url=1724598894637Qa8Y). The dataset will be automatically made public after the publication of the article. The dataset for analyzing the expression level of SDC4 in IPF, COVID-19 related PF, radiation-induced PF and BLM PF is sourced from the GEO database and can be accessed and downloaded from the original studies. Other research data will be shared upon request to the corresponding author.
