# Supplementary material for: Fibrotic lung ECM upregulates SDC4/integrin-αvβ1 interaction and the interfering peptide SDC487-131 and its derivative peptides alleviate pulmonary fibrosis
Source: Regen Biomater. 2025 Jun 16;12:rbaf057. doi: 10.1093/rb/rbaf057 (PMC12313019; doi:10.1093/rb/rbaf057)
Supplement: rbaf057_Supplementary_Data [file rbaf057_supplementary_data.zip › supplementary Methods.docx]

**Fibrotic lung ECM upregulates SDC4 / Integrin-αVβ1 interaction and the interfering peptide SDC4_87-131_ and its derivative peptides alleviate pulmonary fibrosis**

**Supplementary METHODS**

**1. Conditions set for MS/MS data analysis**

The peptides were subjected to capillary source processing and timsTOF Pro (Bruker Daltonics) mass spectrometry analysis. The resulting MS/MS data were processed using MaxQuant search engine (v.1.6.15.0). Tandem mass spectra were searched against the mouse SwissProt database (17089 entries) concatenated with reverse decoy database. Trypsin/P was specified as cleavage enzyme allowing up to 2 missing cleavages. The mass tolerance for precursor ions was set as 20 ppm in first search and 5 ppm in main search, and the mass tolerance for fragment ions was set as 0.02 Da. Carbamidomethyl on Cys was specified as fixed modification, and acetylation on protein N-terminal and oxidation on Met were specified as variable modifications. FDR was adjusted to < 1%.

**2. Blood test of mice**

At the termination of the experiment, the mice were euthanized and their lung tissues were taken after the eyeballs were removed and blood was collected. Within 2 hours after blood collection, the blood samples were tested on the URIT-2900VetPLUS automatic animal blood cell analyzer.

**3. HE staining and Masson staining**

Lung tissue samples were paraffin-embedded, then sliced and dewaxed, and HE and Masson staining were performed respectively. After dehydration and sealing, images were collected and analyzed using a microscope.

**4. Hydroxyproline assay**

100 mg of lung tissue was accurately weighed and processed according to the instructions of the hydroxyproline assay kit (Nanjing Jiancheng, Nanjing, China), and the absorbance was measured at a wavelength of 550 nm. The data were expressed as micrograms (µg) of hydroxyproline per mg of dry lung tissue.

**5. Western blot**

20 mg of lung tissue was extracted with RIPA lysis buffer (Roche International Ltd., Bermuda, USA), and the protein concentration was determined using a BCA protein detection kit (Roche). Protein samples were separated by 10% sodium dodecyl sulfate polyacrylamide gel (SDS-PAGE) electrophoresis and transferred to a PVDF membrane. After blocking, the PVDF membrane was incubated with primary antibodies for α-SMA, Integrin-αV, SDC4, Integrin-β1, p-Smad3, FAK, and p-FAK (CST, #19245, #4711, #12236S, #34971, #9520S, #3285S, and #3284S) at 4°C overnight, followed by incubation with secondary antibodies (CST, #7074P2), development with ECL reagent (SAB), and acquisition of images with a chemiluminescence imaging system (ChemiScope 5600).

**6. Immunofluorescence**

Paraffin sections of lung tissue were dewaxed and boiled in sodium citrate buffer (Servicebio, #G1201-1L) for antigen retrieval. Sections were incubated with primary antibodies against Fibronectin, α-SMA, Syndeca-4, Integrin-αV, p-FAK, or Vinculin overnight at 4°C and then incubated with matching secondary antibodies for 2 hours at room temperature. Nuclei were counterstained with DAPI and photographed and visualized using a Leica SP5 confocal microscope (Leica Biosystems, Germany).

**7. RT-PCR**

Total RNA was extracted from cells or lung tissue (10 mg) using Trizol reagent, and mRNA was reverse transcribed into cDNA using HiScript® II QRT SuperMix for qPCR (Vazyme, #R223-01). Real-time fluorescence quantitative PCR (RT-PCR) was performed using Hieff® qPCR SYB Green Master Mix (YEASEN, #11201ES08) to obtain the Ct value. The 2^−ΔΔCt^ method was used to obtain the relative expression level of mRNA in each group. Primers (Table 1) were purchased from Sangon Biotech.

**Table S1. Primers**

| **Protein** | | **Sequences** | **Number of bases** |
| --- | --- | --- | --- |
| Collagen I | F | CAGTGGCGGTTATGACTTCAG | 21 |
|  | R | GCTGCGGATGTTCTCAATCT | 20 |
| Collagen III | F | TCCTGAAGATGTCGTTGATGTG | 22 |
|  | R | TGTGTAAGTGAATTTGCTGTTTCC | 24 |
| Fibronectin | F | CAGGGCAGCAACATCTTTGA | 20 |
|  | R | CACGATCAGAGCTGCCAAGAC | 21 |
| α-SMA | F | CTGGAGTCAAGCCAGACACA | 20 |
|  | R | CGAGGTGACAGAGACCACAA | 20 |
| TGF-β1 | F | GCGGACTACTATGCTAAAGAGG | 22 |
|  | R | CCGAATGTCTGACGTATTGAAGA | 23 |
| TNF-α | F | GCCGATGGGTTGTACCTTGT | 20 |
|  | R | TCTTGACGGCAGAGAGGAGG | 20 |
| IL-1β | F | GAAATGCCACCTTTTGACAGTG | 22 |
|  | R | TGGATGCTCTCATCAGGACAG | 21 |
| IL-6 | F | TCATTTGCCGAAGAGCCCTG | 20 |
|  | R | GAAGTAGGGAAGGCCGTGG | 19 |
| GAPDH | F | GTGGAGTCATACTGGAACATGTAG | 24 |
|  | R | AATGGTGAAGGTCGGTGTG | 19 |

**8. SDC4 knockdown by shRNA**

The lentivirus loading shRNA-SDC4 was designed and packaged by Hanheng Biotechnology Co., Ltd. NIH3T3 cells was transfected and puromycin was added to screen out stable transgenic strains. Sh-SDC4-2 was used in subsequent experiments for its best knockdown effect (more than 50%). See Tab.2 for the sequences of sh-Ctrl and sh-SDC4.

**Table S2. sh-SDC4 plasmid sequence**

| **Name** | **Sequences** |
| --- | --- |
| sh-SDC4 | 5ˈ- GCG GCG TGG TAG GCA TCC TCT TTG CCG TT-3ˈ |
| sh-Ctrl | 5ˈ- GCA CTA CCA GAG CTA ACT CAG ATA GTA CT-3ˈ |

**9. Flow cytometry analysis**

The single cell suspension of mouse lung tissue was obtained by grinding. After the remaining red blood cells were lysed with ACK Lysis Buffer (TBD, NH4CL2009), 1×10^6^ cells/100 μL of cells were added with Fixable Viability Stain 780 (BD, #565388). After incubation at room temperature in the dark for 10-15 min, the suspension was washed twice with PBS containing 1%-2% FBS. The FCR antibody TruStain FcX™ PLUS (BD, #156603) was added to the cell suspension at a ratio of 0.25 μL:100 μL and blocked on ice for 10 min. Then, the antibody was incubated at an appropriate concentration and time according to the manufacturer's instructions. Antibodies include CD45 (Biolegend, #157619), CD140a (Thermo Fisher, #25-1401-82), SDC-4 (Sino Biological, #50726-R112-P), SP-C (Bioss, #bs-10067R-FITC) and Integrin-β1 (9Eg7, BD, 553715), and AF647 anti-Rat (CST, #4418S) was used as the secondary antibody. After incubation, cells were washed and resuspended, and detected using LSRFortessaX-20 (BD).

**10. FN-coating cell adhesion assay**

10 μg/mL Fibronectin (Merck, #10838039001) was dissolved in serum-free DMEM medium and dropped into a 96-well plate, 50 μL per well. The plate was incubated at 4 °C overnight. After blocking with 3% bovine serum albumin at room temperature for 30 min, 4×10^4^ cells were inoculated into each well. After incubation for 30 min, the cells were washed, fixed, and stained with crystal violet (5 mg/mL) for 30 min. Then after the crystal violet was dissolved, it was detected using an ELISA reader at 595 nm.

**11. Single-cell RNA sequencing analysis**

Single-cell RNA sequencing datasets of lung tissues from IPF patients (GSE213017), COVID-19 patients (GSE149878), mouse radiation-induced PF (GSE211713) and mouse BLM-induced PF (GSE210341) were obtained from the Gene Expression Omnibus (GEO) database. Analyses were conducted using R software (version 4.4.1). Cells with >5000 or <500 expressed genes or mitochondrial gene content >25% were filtered. Normalization and identification of 2000 highly variable genes were performed using Seurat package (version 5.0.0). Batch effects were corrected using "RunHarmony" in Harmony package. UMAP was done with a resolution of 0.5 to visualize clusters. To annotate cell types within each cluster, the Wilcoxon rank-sum test was applied via the "FindAllMarkers" function in Seurat. Cell type assignment was based on canonical marker genes from recent single-cell RNA-seq studies [1, 2]. Fibroblasts and unannotated cells were isolated, and SDC4 gene expression was visualized with violin plots. Statistical significance of SDC4 expression was evaluated using the Wilcoxon rank-sum test.

**12. In Vivo and Ex vivo fluorescence imaging observation of the distribution of CS-9 in lung tissue**

CS-9 was labeled with Cy5.5-NHS ester. Thoroughly mix the Cy5.5-NHS dye with a concentration of 10 mg/mL CS-9 (make the molar amount of the dye / the molar amount of CS-9 > 3 to ensure sufficient labeling of the polypeptide), and place the mixture on a shaker at 4°C for a reaction lasting 2-4 hours. Remove the excessive Cy5.5-NHS by dialysis, and lyophilize the labeled CS-9 to obtain the purified labeled polypeptide. Then the labeled CS-9 was administered via oropharyngeal inhalation and subjected to fluorescence imaging using PerkinElmer IVIS SPECTRUM In Vivo Imaging System. After anesthetizing the mice, administer the labeled CS-9 via oropharyngeal instillation at a dose of 350 μg/kg, and use the same volume of normal saline as the control. The small animal fluorescence imaging system was used to observe the fluorescence imaging of mice at 0h, 0.5h, 1h, 2h, 4h, and 6h after administration. The mice were sacrificed and the intact lung tissues were removed to directly observe the accumulation of the drug in the lungs.

**References**

1. Habermann AC, Gutierrez AJ, Bui LT, Yahn SL, Winters NI, Calvi CL, Peter L, Chung MI, Taylor CJ, Jetter C *et al*: **Single-cell RNA sequencing reveals profibrotic roles of distinct epithelial and mesenchymal lineages in pulmonary fibrosis**. *Sci Adv* 2020, **6**(28):eaba1972.

2. Liu X, Qin X, Qin H, Jia C, Yuan Y, Sun T, Chen B, Chen C, Zhang H: **Characterization of the heterogeneity of endothelial cells in bleomycin-induced lung fibrosis using single-cell RNA sequencing**. *Angiogenesis* 2021, **24**(4):809-821.
