# Supplementary material for: Fibrotic lung ECM upregulates SDC4/integrin-αvβ1 interaction and the interfering peptide SDC487-131 and its derivative peptides alleviate pulmonary fibrosis
Source: Regen Biomater. 2025 Jun 16;12:rbaf057. doi: 10.1093/rb/rbaf057 (PMC12313019; doi:10.1093/rb/rbaf057)
Supplement: rbaf057_Supplementary_Data [file rbaf057_supplementary_data.zip › Supplemental Tables.docx]

**Fibrotic lung ECM upregulates SDC4 / Integrin-αVβ1 interaction and the interfering peptide SDC4_87-131_ and its derivative peptides alleviate pulmonary fibrosis**

**Supplemental Tables**

**Table S1. Binding energy of two stable binding modes between SDC4_87-131_ and the extracellular region of integrin-αV**

| **Name** | **MMGBSA (kcal/mol)** | **MMPBSA (kcal/mol)** |
| --- | --- | --- |
| Model 1 | -33.19 | 51.71 |
| Model 2 | -88.99 | -3.86 |

**Table S2. Comparison of the stability and binding energy of the binding modes of the three polypeptide chains and integrin-αV**

| **Peptides** | **Models’ similarity** | **Binding energy (MMGBSA)** | **Binding energy (MMPBSA)** |
| --- | --- | --- | --- |
| SDC4_87-131_ | 0.946 | -96.26 | -10.26 |
| SDC1_93-120_ | 0.936 | -4.79 | 33.18 |
| SDC1_210-240_ | 0.935 | -87.49 | 14.67 |

**Table S3. Sequences of SDC4_87-131_ and designed peptides**

| **No.** | **Sequence** | **pTM** |
| --- | --- | --- |
| SDC4_87-131_ | NHIPERAGSGSQVPTEPKKLEENEVIPKRISPVEESEDVSNKVSM | 0.63 |
| d01 | NHIPERAGSGSQVPTEPKKLEEIPKRISPVEESEDVSNKVSM | 0.59 |
| d02 | NHIPERAGSGSQVPTEPKKLSPVEESEDVSNKVSM | 0.58 |
| d03 | NHIPERAVPTEPKKLEENEVIPKRISPVEESEDVSNKVSM | 0.62 |
| d04 (CS-1) | NHIPERAGSGSQVPTEPKKLSPVEESEDVSNKVSMTTNVT | 0.66 |
| d05 | NHIPERAVPTEPKKLEENEVIPKRISPVEESEDVSNKVSMTTNVT | 0.64 |
| d06 | NHIPERAVPTEPKKLSPVEESEDVSNKVSMTTNVT | 0.63 |
| d07 (CS-2) | NHIPERAGSGSQVPTEPKKLSPVEESEDRSNKVSM | 0.66 |
| d08 (CS-3) | NHIPERAGSGSQVPTEPKKLSPVEESEDWSNKVSM | 0.66 |
| d09 (CS-4) | NHIPERAVPTEPKKLSPVEESEDITNSTLVTTNVT | 0.67 |
| d10 (CS-8) | NHIPERAVPTEPKKLSPVEESEDITNSTLV | 0.67 |
| d11 | ELEELPLLKTSQVPTEPKKLSPVEESEDVSNKVSMTTNVT | 0.67 |
| d12 (CS-5) | LPELPPEAKTSQVPTEPKKLSPVEESEDVSNKVSMTTNVT | 0.69 |
| d13 | LVEEPRAGSGSQVPTEPKKLSPVEESEDVSNKVSMTTNVT | 0.68 |
| d14 (CS-6) | AVAEPRAGSGSQVPTEPKKLSPVEESEDVSNKVSMTTNVT | 0.69 |
| d15 (CS-7) | NHIPEKKEKTSQVPTEPKKLSPVEESEDVSNKVSMTTNVT | 0.68 |
| d16 | NHIPELKAKTSQVPTEPKKLSPVEESEDVSNKVSMTTNVT | 0.67 |
| d17 | NHIPERAGSGSQVPTEPKKLSPVEESEDRWSNKVSM | 0.60 |
| d18 | LDIPPPEEPESQVPTEPKKLSPVEESEDRSNKVSM | 0.64 |
| d19 | LEPEPPEEPESQVPTEPKKLSPVEESEDRSNKVSM | 0.60 |
| d20 | LDPPPRAGSGSQVPTEPKKLSPVEESEDRSNKVSM | 0.65 |
| d21 | IEIPPRAGSGSQVPTEPKKLSPVEESEDRSNKVSM | 0.56 |
| d22 | NHIPEKKEEKSQVPTEPKKLSPVEESEDRSNKVSM | 0.64 |
| d23 | NHIPEASEPRSQVPTEPKKLSPVEESEDRSNKVSM | 0.61 |
| d24 | GAADPTEEEQEPKKLSPVEESEDITNSTLV | 0.61 |
| d25 | GSAPATVEEQEPKKLSPVEESEDITNSTLV | 0.65 |
| d26(CS-10) | GSADPRAVPTEPKKLSPVEESEDITNSTLV | 0.66 |
| d27 | GAADPRAVPTEPKKLSPVEESEDITNSTLV | 0.61 |
| d28 | NHIPETEEDTEPKKLSPVEESEDITNSTLV | 0.65 |
| d29 (CS-9) | NHIPETVEDPEPKKLSPVEESEDITNSTLV | 0.71 |
| d30 | NHIPERPTEPKKLSPVEESEDITNSTLV | 0.62 |

**Table S4. Comparison of amino acid sequences of SDC4_87-131_ and designed peptides**

| **Peptides** | **Sequence** | **Length (aa)** |
| --- | --- | --- |
| SDC4_87-131_ | Ac-NHIPERAGSGSQVPTEPKKLEENEVIPKRISPVEESEDVSNKVSM-NH_2_ | 45 |
| CS-1 | Ac-NHIPERAGSGSQVPTEPKKL----------SPVEESEDVSNKVSM--TTNVT-NH_2_ | 40 |
| CS-2 | Ac-NHIPERAGSGSQVPTEPKKL----------SPVEESEDRSNKVSM-NH_2_ | 35 |
| CS-3 | Ac-NHIPERAGSGSQVPTEPKKL----------SPVEESEDWSNKVSM-NH_2_ | 35 |
| CS-4 | Ac-NHIPERA----------VPTEPKKL----------SPVEESED--ITNSTLVTTNVT-NH_2_ | 35 |
| CS-5 | AVAEP--RAGSGSQVPTEPKKL----------SPVEESEDVSNKVSM--TTNVT | 40 |
| CS-6 | LPELPPEAKT--SQVPTEPKKL----------SPVEESEDVSNKVSM--TTNVT | 40 |
| CS-7 | NHIPE-KKEKT-SQVPTEPKKL----------SPVEESEDVSNKVSM--TTNVT | 40 |
| CS-8 | Ac-NHIPERA----------VPTEPKKL----------SPVEESED--ITNSTLV-NH_2_ | 30 |
| CS-9 | Ac-NHIPE-----TVEDP-----EPKKL----------SPVEESED--ITNSTLV-NH_2_ | 30 |
| CS-10 | Ac-GSADPRA---------VPTEPKKL----------SPVEESED--ITNSTLV-NH_2_ | 30 |

Note: CS-5, CS-6, and CS-7 could not be successfully synthesized, and the other synthetic peptides were modified with N-terminal acetylation and C-terminal amidation.

**Table S5. Binding energy comparison of the docking mode of SDC4_87-131_ and designed peptides with the Calf-1/Calf-2 domain of Integrin-αV extracellular region**

| **Peptides** | **AlphaFold2 predicted**  **structure (pLDDT)** | **MMGBSA (kcal/mol)** | **MMGPSA (kcal/mol)** |
| --- | --- | --- | --- |
| SDC4_87-131_ | 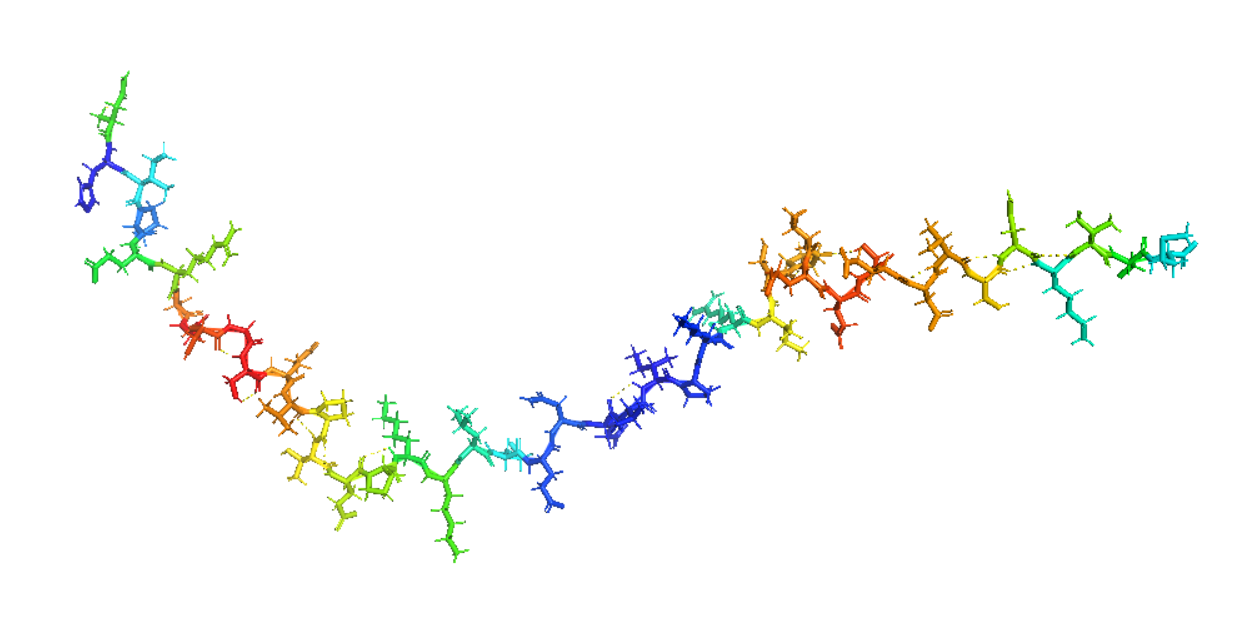 | -88.99 | -3.86 |
| CS-1 | 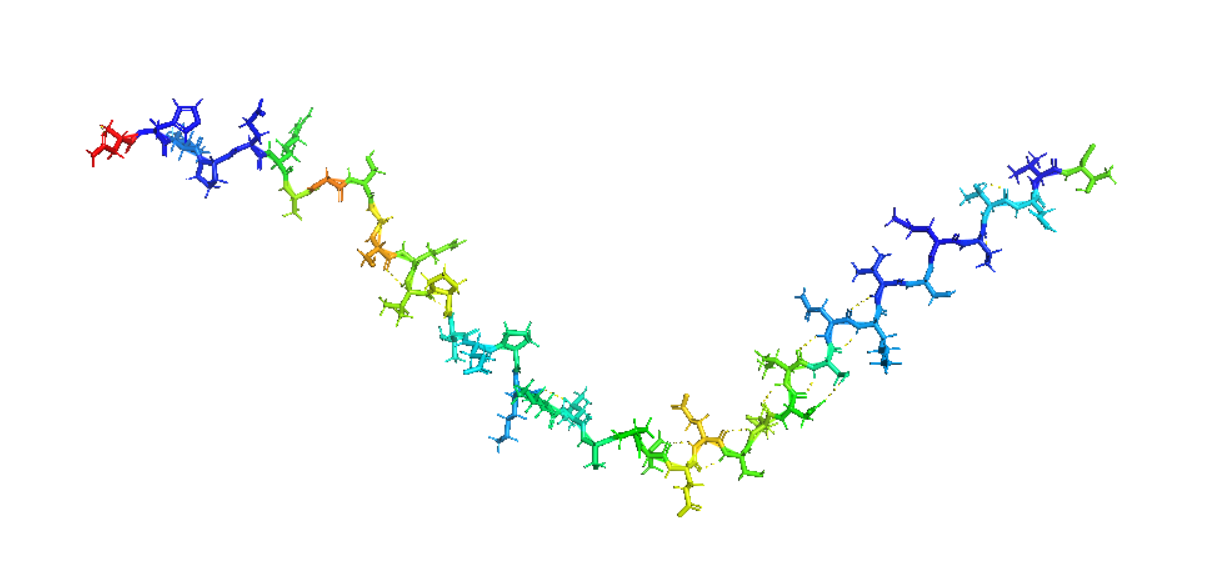 | -105.18 | -36.74 |
| CS-2 | 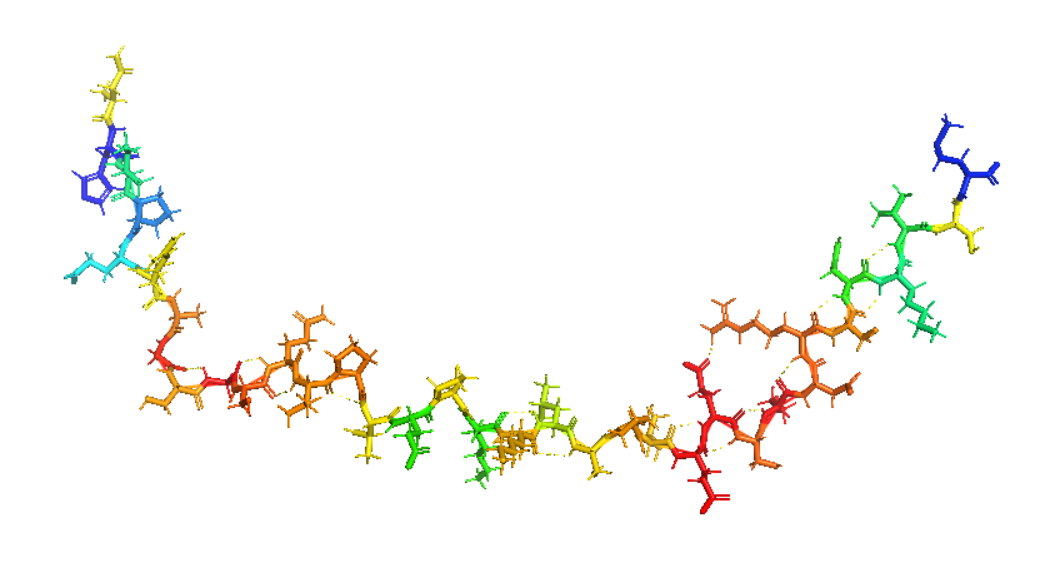 | -86.48 | -1.52 |
| CS-3 | 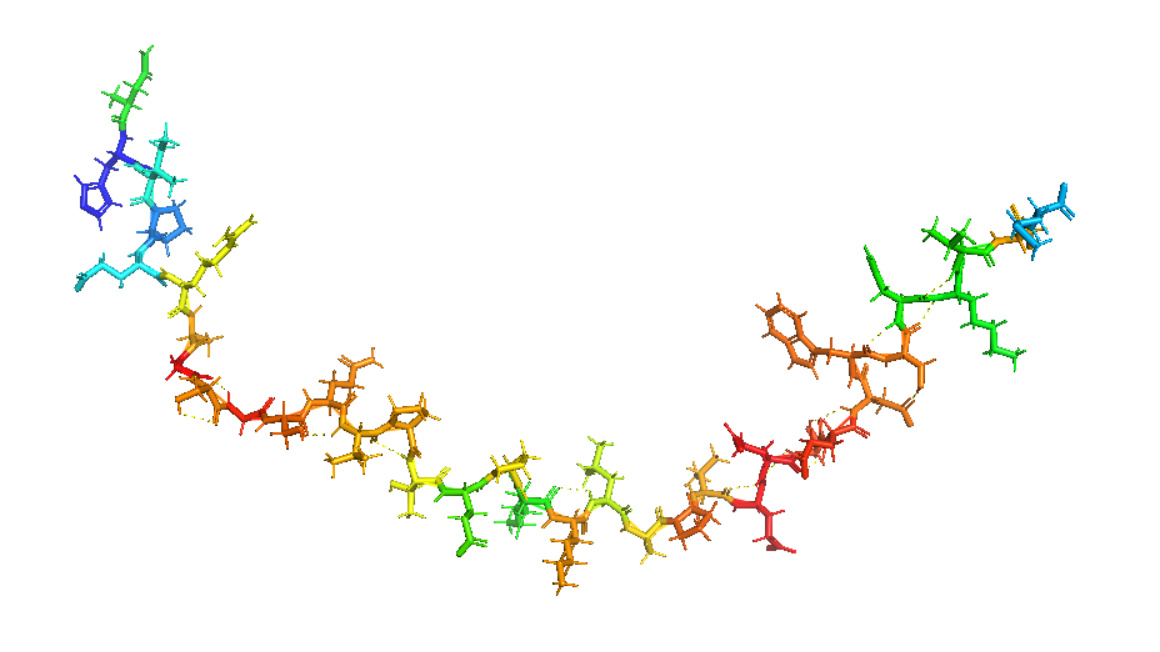 | -10.25 | 16.86 |
| CS-4 | 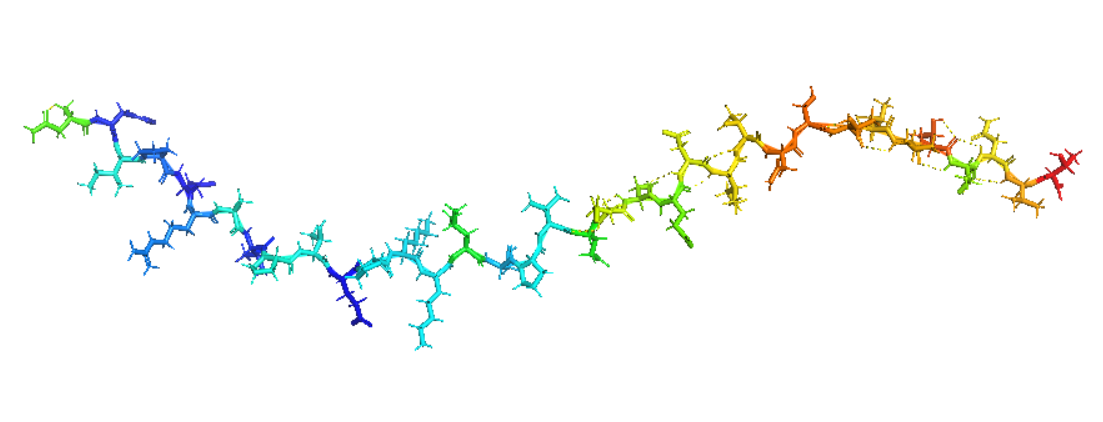 | -112.49 | -28.81 |
| CS-5 | 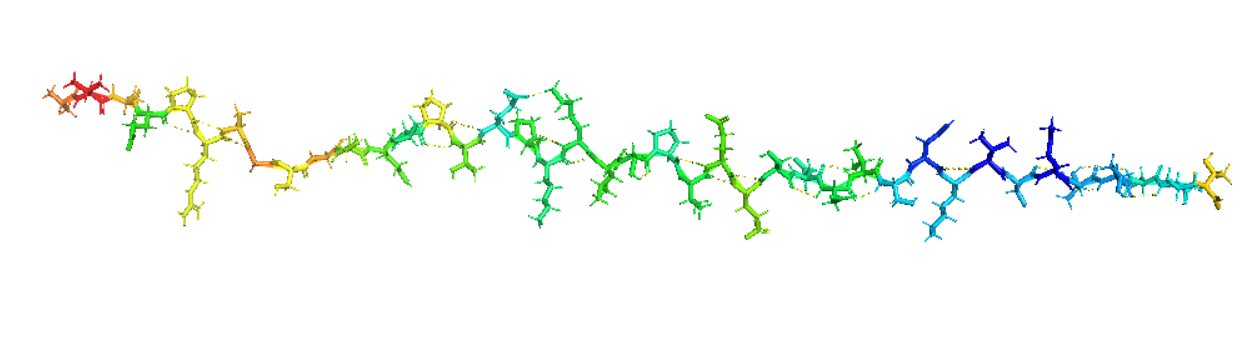 | -113.48 | -8.77 |
| CS-6 | 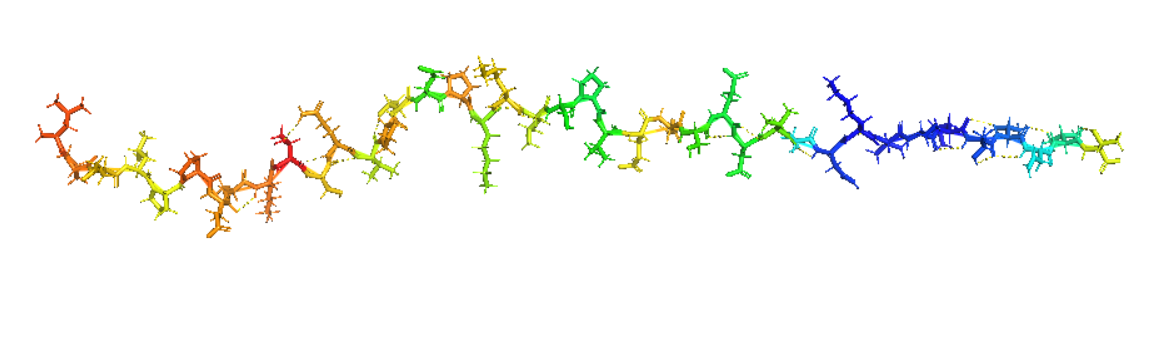 | -112.28 | -31.85 |
| CS-7 | 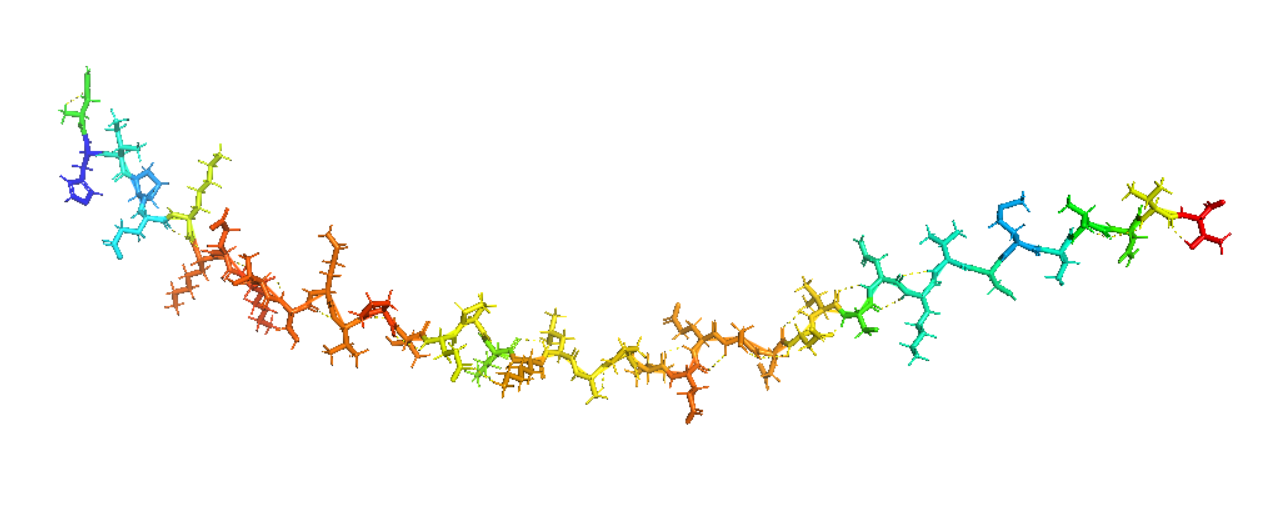 | -109.6 | -21.63 |
| CS-8 | 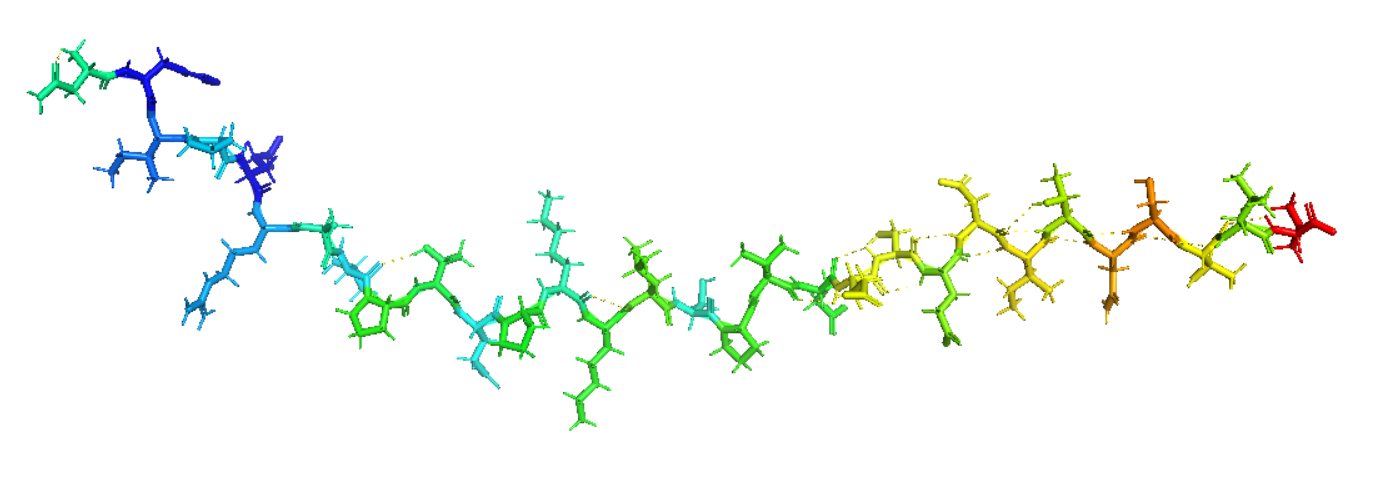 | -109.33 | -18.46 |
| CS-9 | 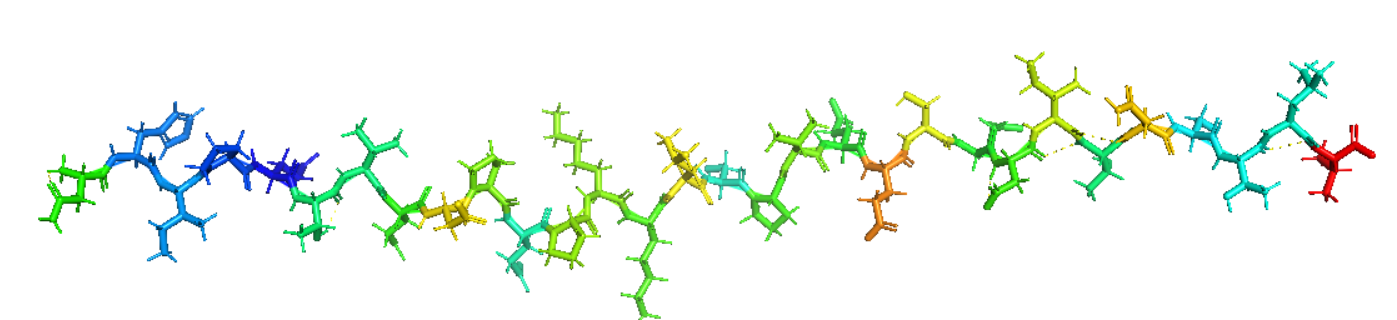 | -123.43 | -36.1 |
| CS-10 | 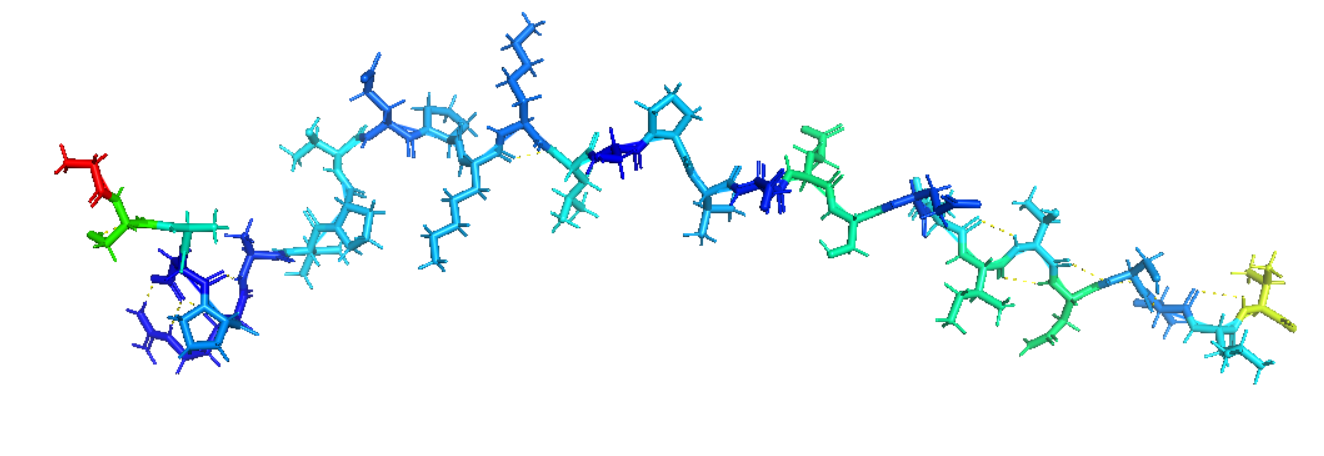 | -96.93 | -6.27 |

Note: CS-5, CS-6, and CS-7 could not be successfully synthesized.
