# Supplementary material for: Fibrotic lung ECM upregulates SDC4/integrin-αvβ1 interaction and the interfering peptide SDC487-131 and its derivative peptides alleviate pulmonary fibrosis
Source: Regen Biomater. 2025 Jun 16;12:rbaf057. doi: 10.1093/rb/rbaf057 (PMC12313019; doi:10.1093/rb/rbaf057)
Supplement: rbaf057_Supplementary_Data [file rbaf057_supplementary_data.zip › Supplemental Figures.docx]

**Fibrotic lung ECM upregulates SDC4 / Integrin-αVβ1 interaction and the interfering peptide SDC4_87-131_ and its derivative peptides alleviate pulmonary fibrosis**

**Supplemental Figures**


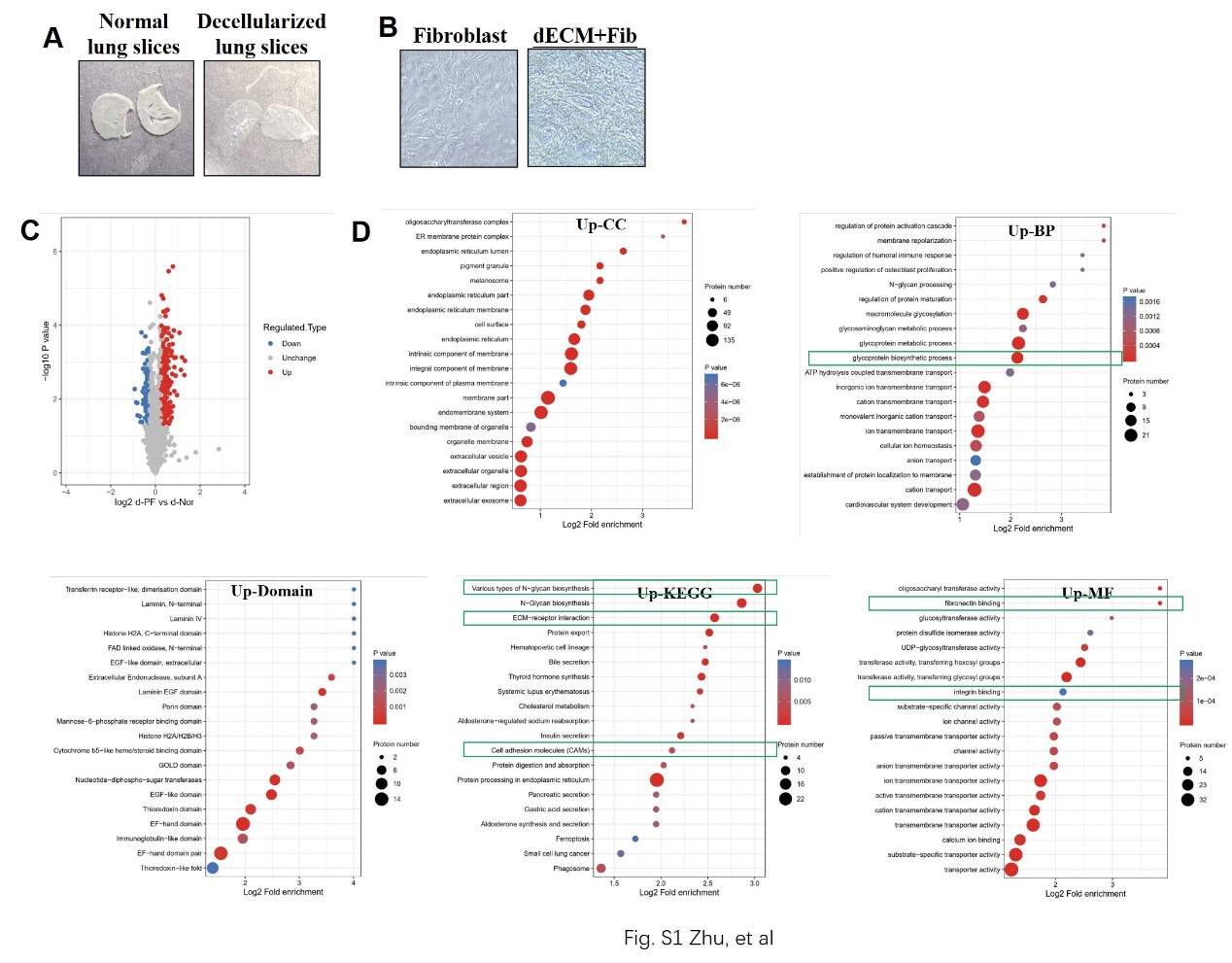


**Figure S1. dECM+Fib three-dimensional culture and proteomic data analysis.**

(**A**) Frozen sections of normal lung tissue and decellularized lung tissue (dECM). (**B**) Fibroblast cultured in petri dishes and on dECM (dECM+Fib). (**C-D**) Fibroblast was seeded on dECM-Nor and dECM-PF, cultured for 48 hours, and cells were collected for proteomic analysis (n=3); (**C**) Volcano plot; (**D**) Enrichment analysis of upregulated genes in dECM-PF group compared with dECM-Nor group.


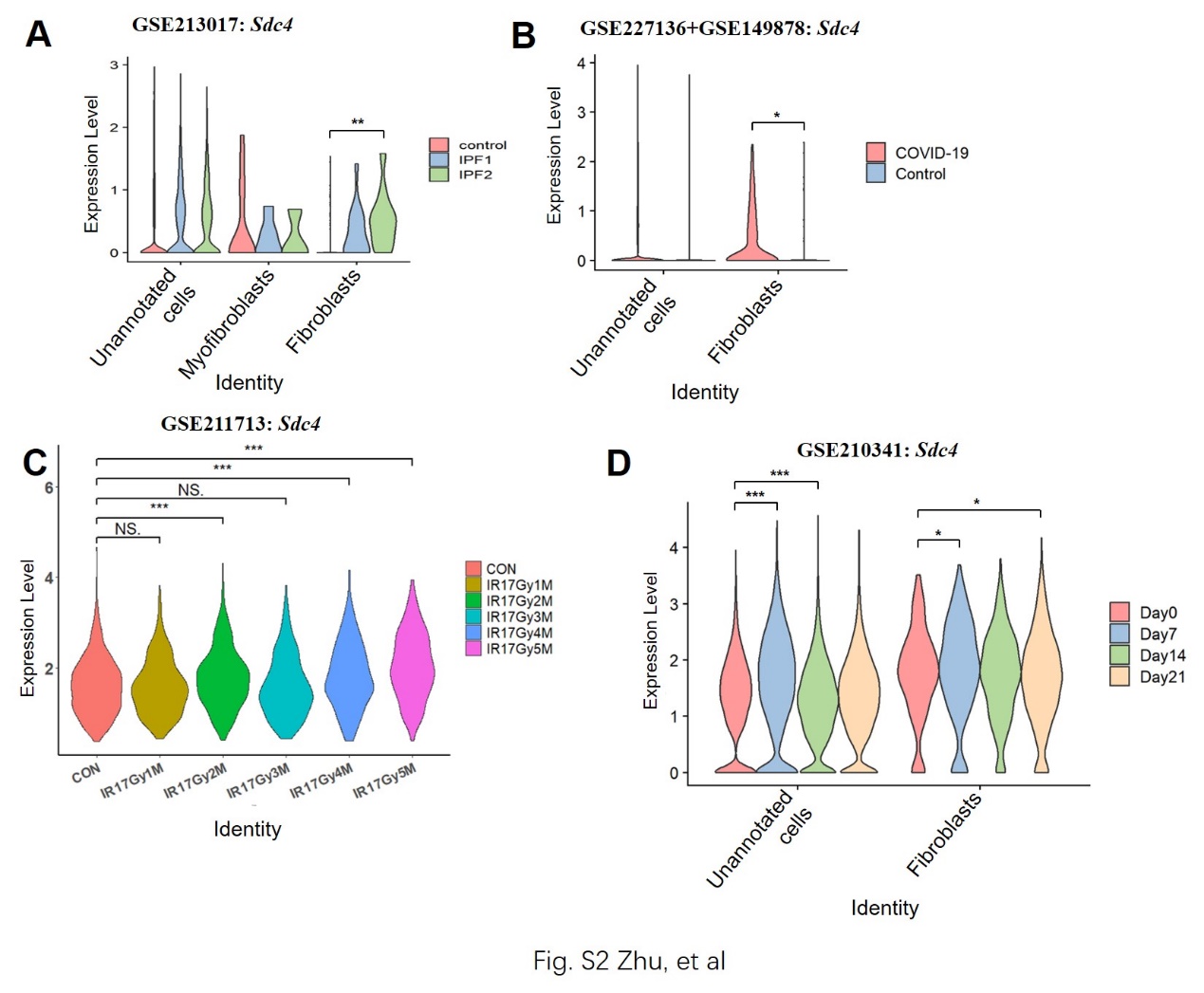


**Figure S2. Analysis of levels of SDC4 mRNA in fibroblasts in single-cell sequencing datasets.**

(**A**) GSE213017, lung tissues from donor (control) and from IPF patients (IPF1 and IPF2) (n=1); (**B**) For analysis, GSE227136 and GSE149878 were merged. GSE227136, lung tissues from healthy individuals, and GSE149878, from COVID-19 patients (n=4); (**C**) GSE211713, fibroblasts in lung tissues from mouse radiation-induced pulmonary fibrosis (n=2); (**D**) GSE210341, lung tissues from mouse BLM-induced pulmonary fibrosis (n=3). Note: *p < 0.05, **p < 0.01, ***p<0.001.


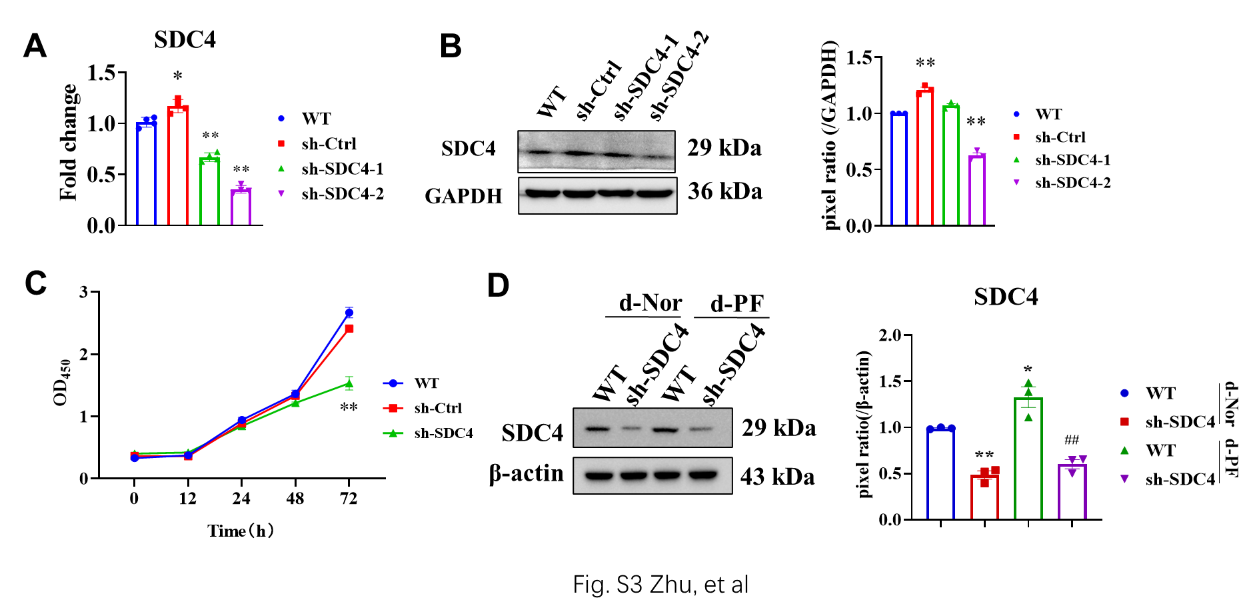


**Figure S3. Knockdown of SDC4 using sh-RNA.**

(**A-C**) NIH3T3 cells were infected with lentivirus carrying sh-SDC4. RT-PCR (**A**) and Western blot (**B**) were used to detect knockdown efficiency (n=3-4), showing that sh-SDC4-2 had a higher knockdown efficiency (about 40%). Subsequent experiments were performed using the sh-SDC4-2 stable knockdown strain; (**C**) CCK-8 assay showed that knockdown of SDC4 could slow down cell proliferation; Note: *p < 0.05, **p < 0.01 vs. WT. (**D**) WT and SDC4 knockdown cells were cultured on d-Nor and d-PF, respectively, and the expression level of SDC4 was detected by Western blot (n=3). Note: *p < 0.05, **p < 0.01 vs. WT in d-Nor group; ##p < 0.01 vs. WT in d-PF group. d-Nor: dECM-Nor; d-PF: dECM-PF; WT: wild type.


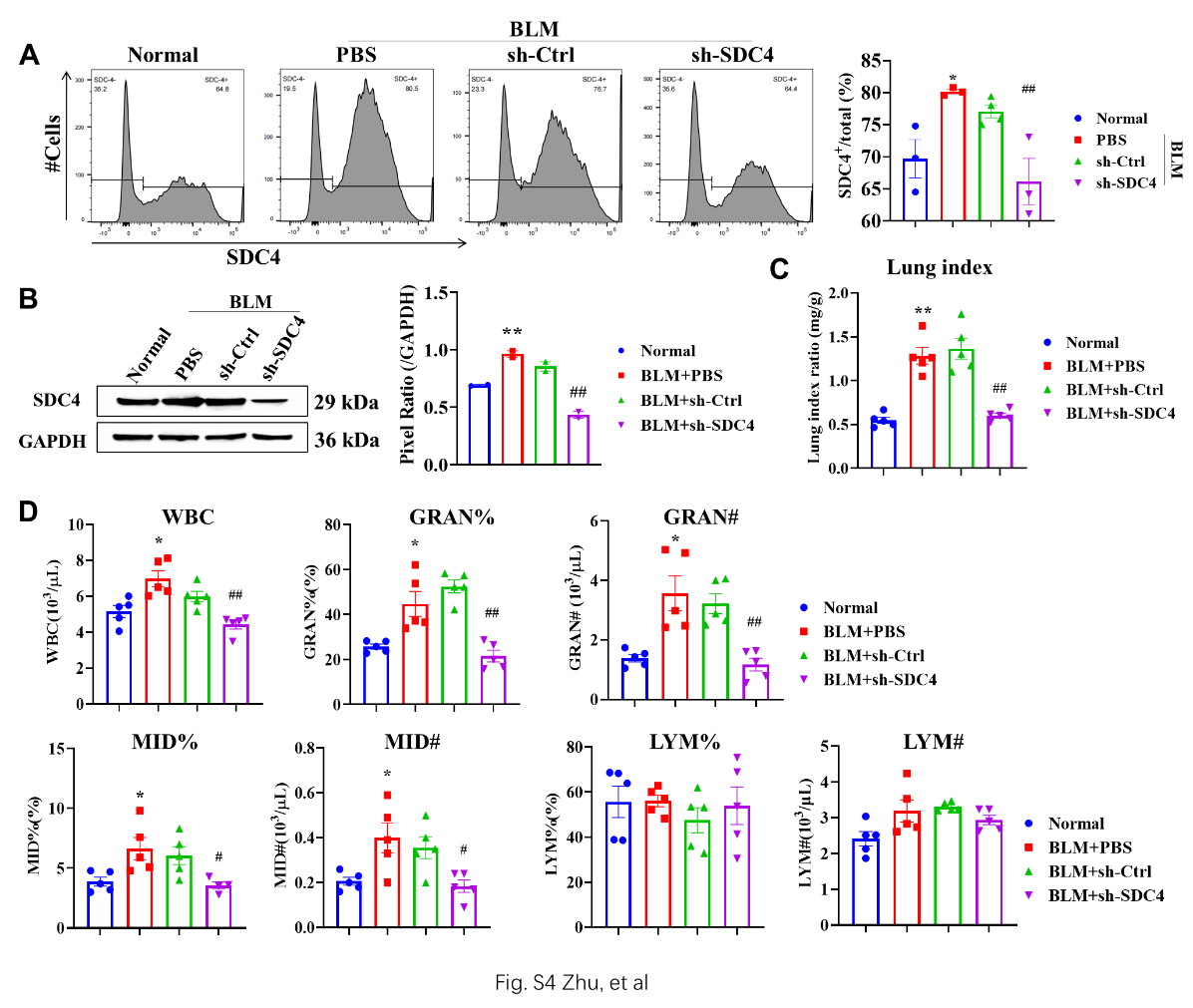


**Figure S4. Knockdown of SDC4 downregulates lung index and white blood cells in BLM-induced lung fibrosis.**

(**A-D**) 1.5×10^7^ TU lentivirus carrying sh-Ctrl or sh-SDC4 was administrated via ITD on day 3 and day 10 post BLM administration, and lung tissue samples were collected on day 21. (**A**) The expression level of SDC4 in lung tissue on 21^st^ day was assessed by flow cytometry (n=3-4). (**B**) The level of SDC4 in lung tissue on 21^st^ day was determined by Western blot (n=3); (**C**) The lung index was calculated and compared (n=5); (**D**) Blood cells were counted using an animal blood analyzer (URIT BH-40Vct, URIT Medical Electronic Co., Ltd., Guilin, China) (n=5). Note: *p < 0.05, **p < 0.01 vs. Normal group; #p < 0.05, ##p < 0.01 vs. BLM (PBS) group; ITD: Inhaled tracheal drip; WBC: white blood cell; GRAN: neutrophil; MID: middle cell; LYM: lymphocyte.


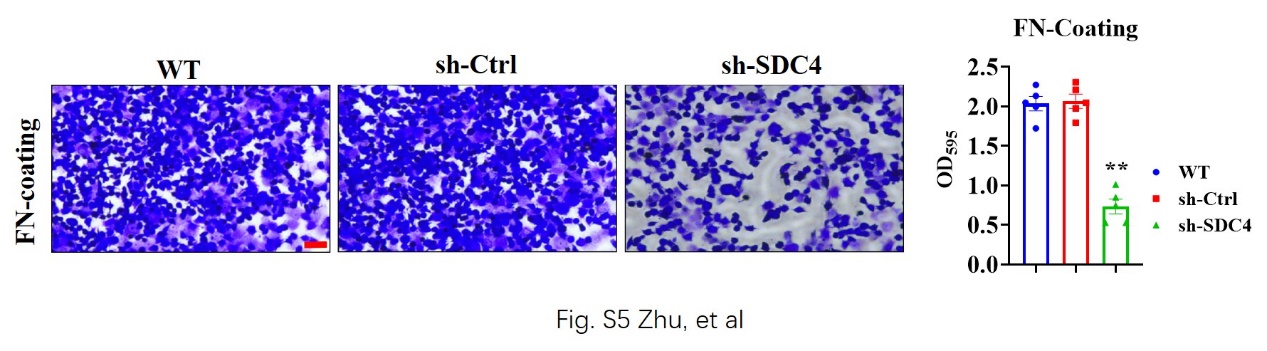


**Figure S5. Knockdown of SDC4 reduces pan-Integrins activation.**

The fibronectin (FN)-coating cell adhesion assay was used to indirectly understand the activation level of pan- Integrins. Cells were seeded on culture plates coated with 10 μg/mL FN, incubated for 30 min, and then stained with crystal violet (scale bar = 25 μm, n=5). Note: **p < 0.01 vs. WT group.


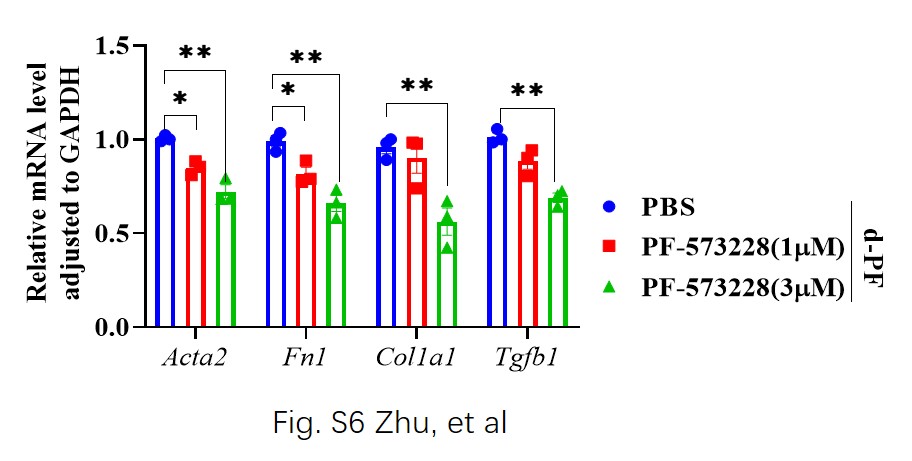


**Figure S6. The FAK inhibitor PF-573228 inhibits dECM-PF-induced fibroblast activation and TGF-β1 synthesis.**

The effect of FAK inhibitor PF-573228 (1μM, 3μM) on the mRNA levels of α-SMA, FN, Col I, and TGF-β1 in fibroblast cultured on d-PF (n=4). Note: **p< 0.01. d-PF: dECM-PF.


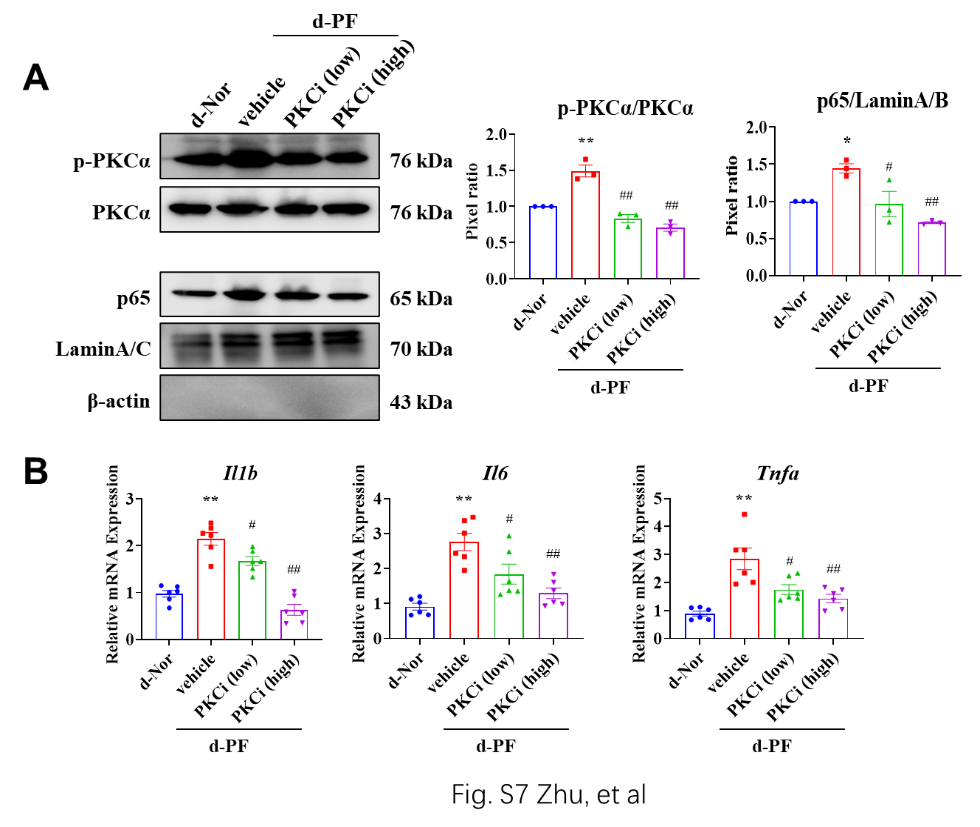


**Figure S7. Inhibition of PKCα can significantly inhibit the nuclear translocation of NFκB and downregulate the levels of inflammatory factors.**

(**A-B**) NIH3T3 cells were seeded on d-Nor and d-PF, respectively, and treated with PKCαinhibitor, PKCiota-IN-2 formic at 10nM (low) and 20nM (high). (**A**) The expression levels of p-PKCα, PKCα and nuclear level of p65 were detected, and the pixel ratio of each protein was analyzed using ImageJ (n=3). (**B**) RT-PCR measured mRNA levels of IL-1β, IL-6, and TNF-α (n=6). Note: *p<0.05, **p < 0.01 vs. d-Nor group; #p<0.05, ##p<0.01 vs. vehicle group.


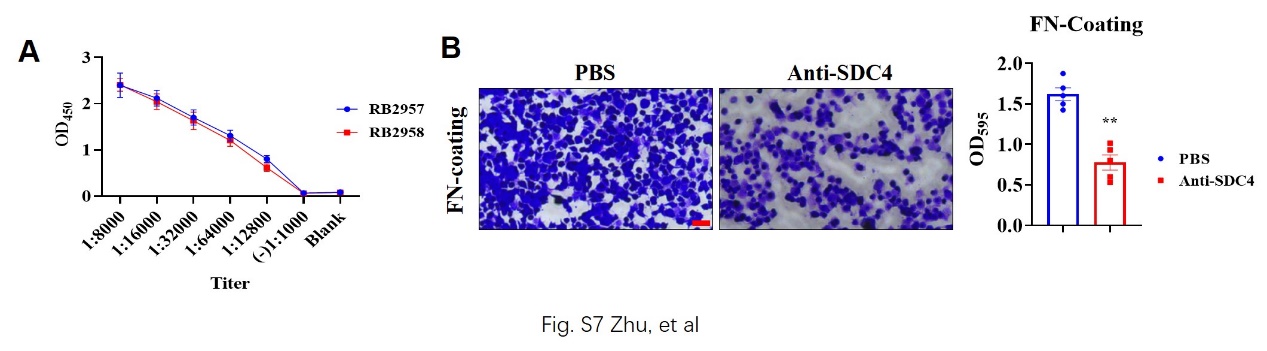


**Figure S8. The polyclonal antibody Anti-SDC4_(93-121)_ inhibits pan-integrin activation.**

(**A**) ELISA result for antigen affinity of purified pAb. Antigen: SDC4_93-121_; RB2957, RB2958: the immunized animal ID; (-): serum from control animal; (**B**) The effect of Anti-SDC4_(93-121)_ 500 ng/ml was evaluated by FN-coating cell adhesion assay (scale bar= 25 μm, n=5). Note: **p < 0.01 vs. PBS group.


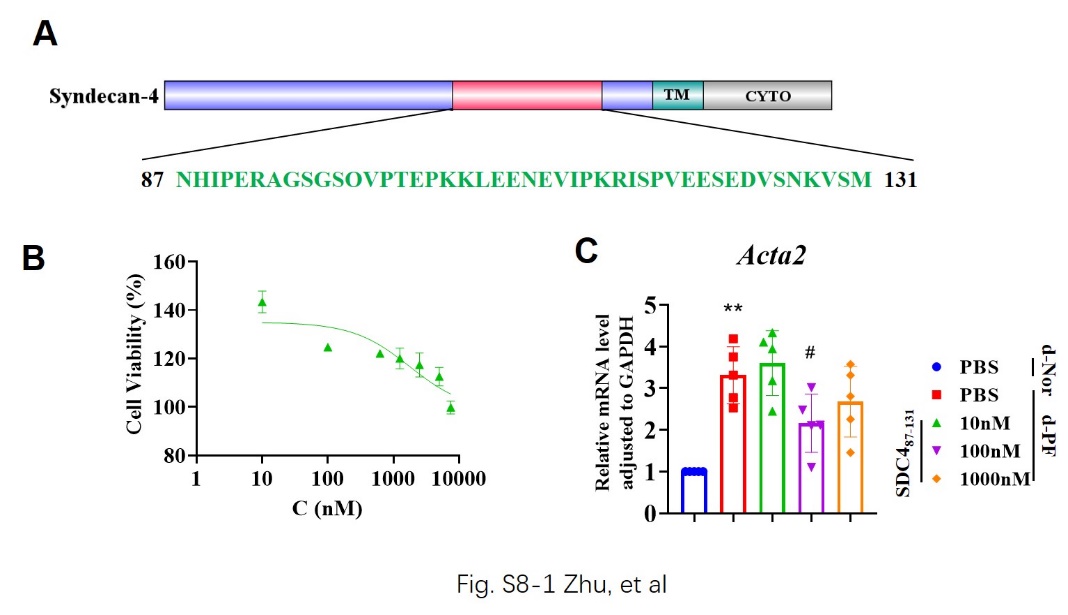


**Figure S9. Effects of SDC4_87-131_ on proliferation and activation of fibroblast.**

(**A**) Schematic diagram of SDC4_87-131_; (**B**) Effect of SDC4_87-131_ and GRGDNP on fibroblast proliferation at 48 h was determined by CCK-8 method (n=3); (**C**) Effect of different concentration of SDC4_87-131_ on fibroblast activation induced by d-PF for 48 h (n=5). Note: **p < 0.01 vs. PBS in d-Nor group; #p < 0.05 vs. PBS in d-PF group. d-Nor: dECM-Nor; d-PF: dECM-PF.


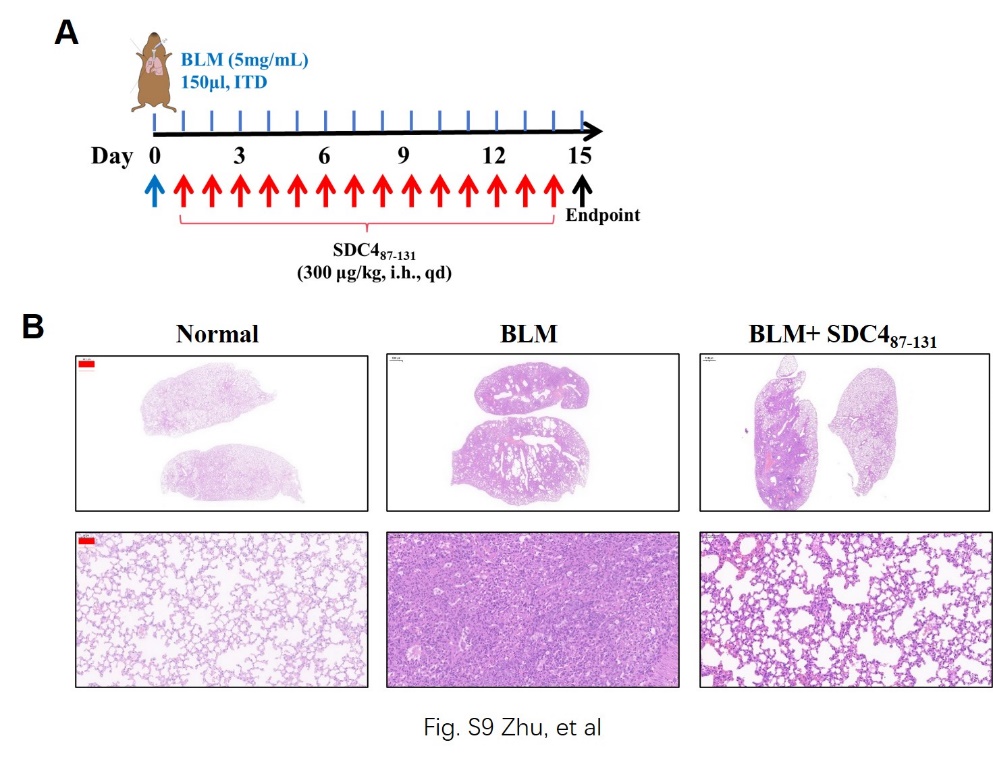


**Figure S10. SDC4_87-131_ can alleviate BLM lung injury in the subacute stage.**

(**A**) Flow chart of animal experiments. SDC4_87-131_ (300 μg/kg, i.h., qd) was administered starting 24 h after BLM modeling, and the experiment ended on the 15^th^ day; (**B**) The pathological changes at the subacute stage of BLM lung injury were observed by HE staining (scale bar = 1000 μm, 50μm). The above results show that SDC4_87-131_ (300μg/kg, i.h., qd) is effective. To achieve better results, we used intratracheal instillation of SDC4_87-131_ (1mg/kg, q3d).


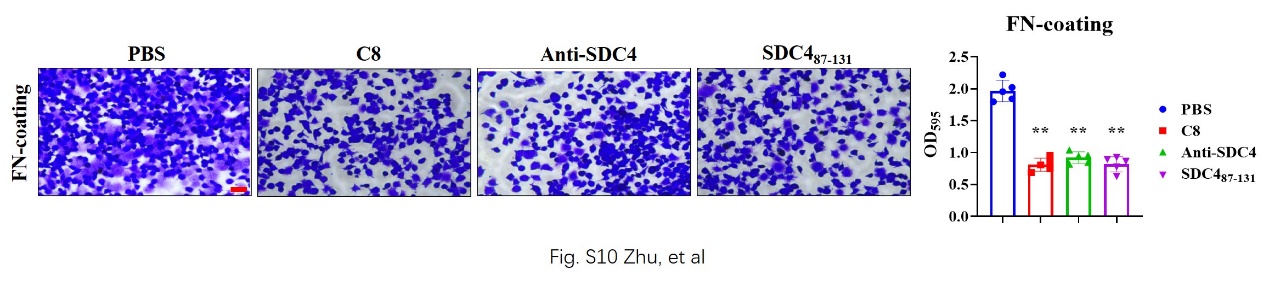


**Figure S11. SDC4_87-131_ inhibits pan-integrin activation.**

The effect of SDC4_87-131_ 100 nM was evaluated by FN-coating cell adhesion assay (scale bar= 25 μm, n=5). Note: **p < 0.01 vs. PBS group.


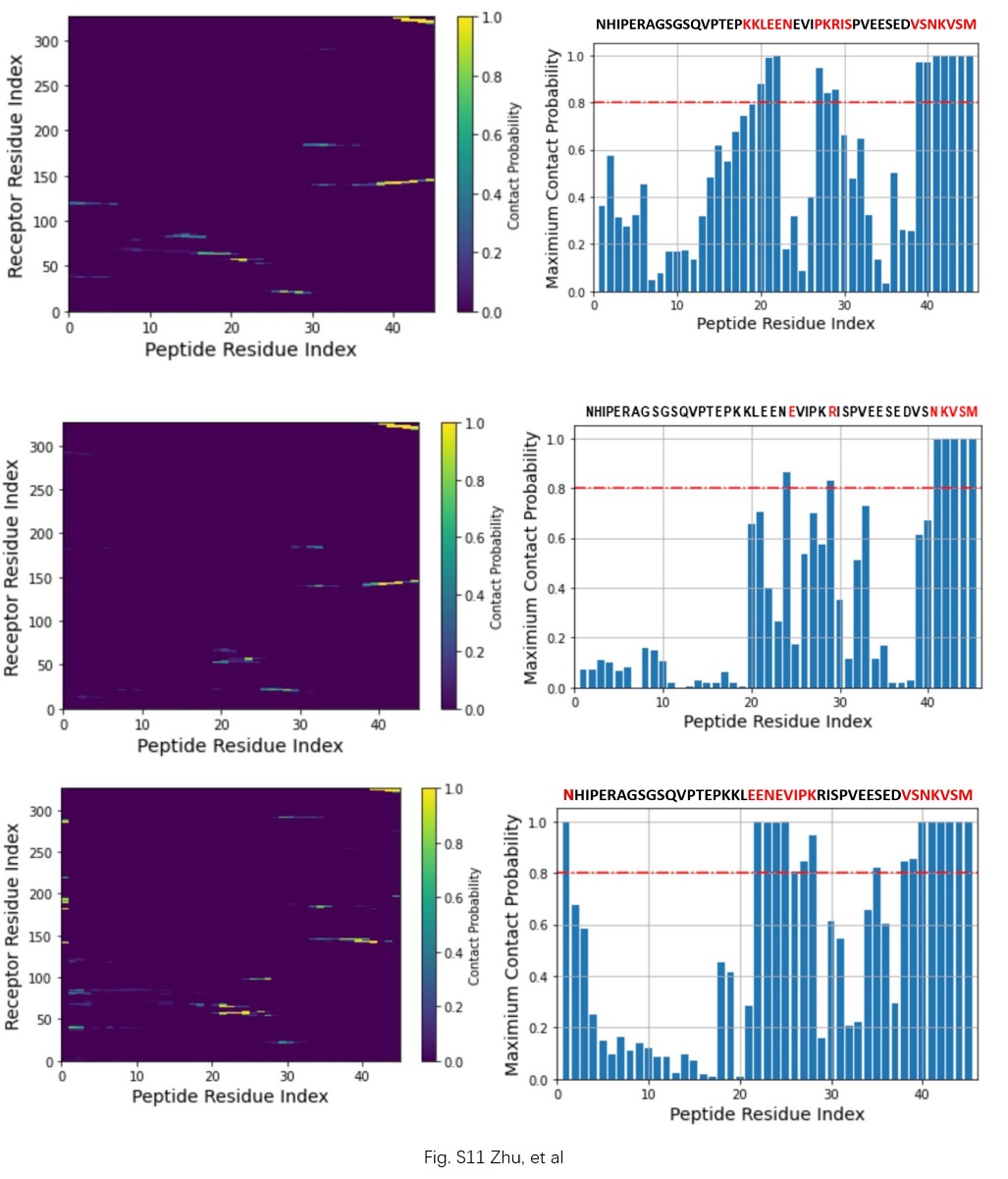


**Figure S12. Confidence analysis of model 2 of the stable binding mode between SDC4_87-131_ and Integrin-αV.**

The results of TOP 3 showed that the binding sites with confidence levels above 0.8 (marked in red) were mainly located at the C-terminus, middle segment, and individual N-terminal amino acids of SDC4_87-131_.


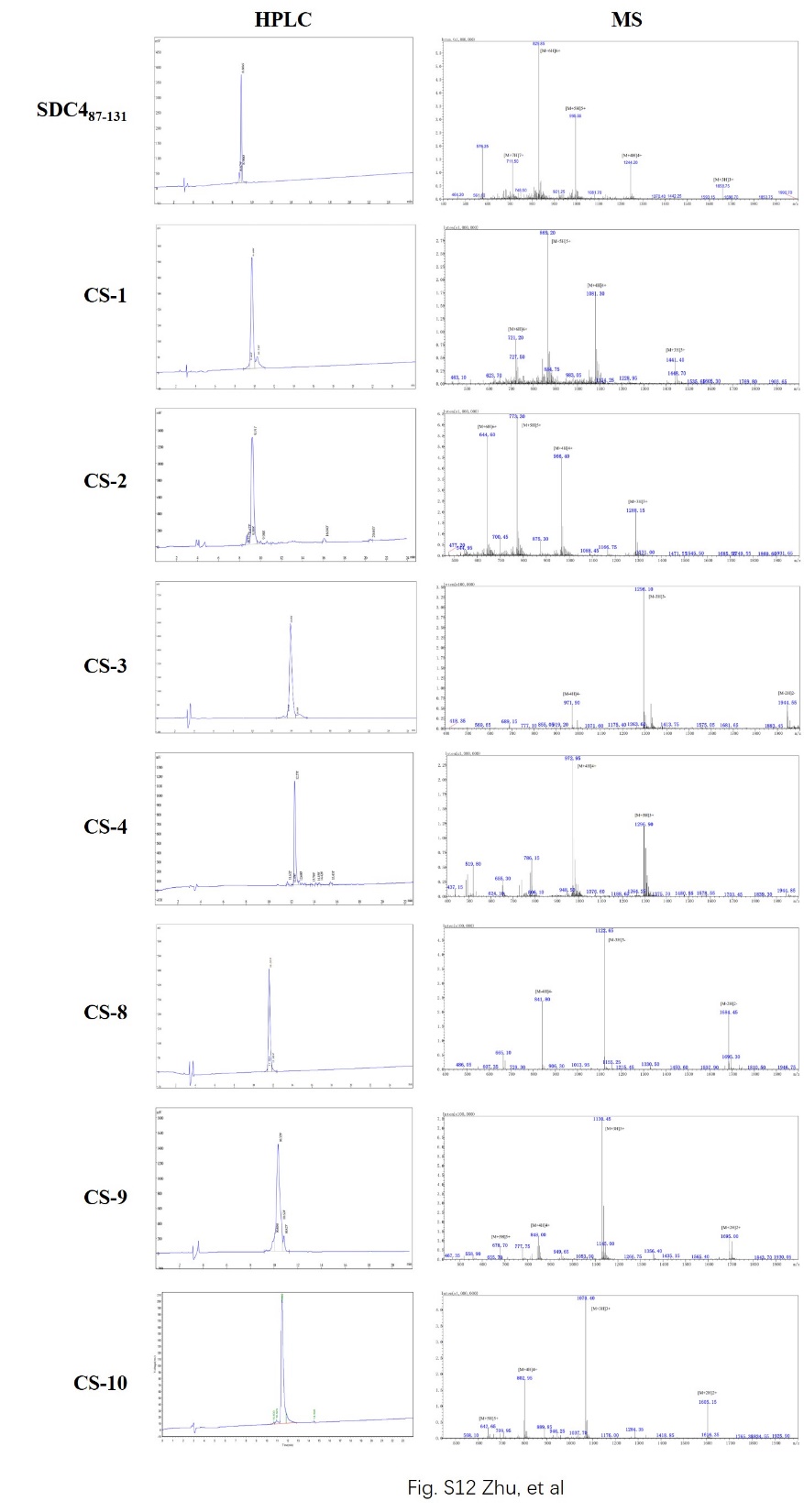


**Figure S13. HPLC and MS spectra of synthesized SDC4_87-131_ and designed peptides.**


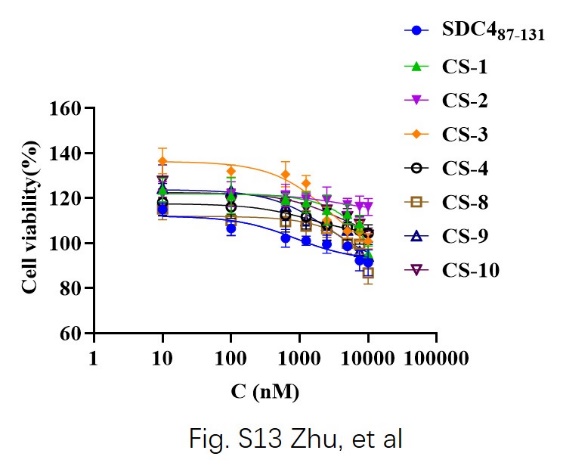


**Figure S14. The viability of fibroblast after treated with peptides for 48 h was detected by CCK-8 assay.**


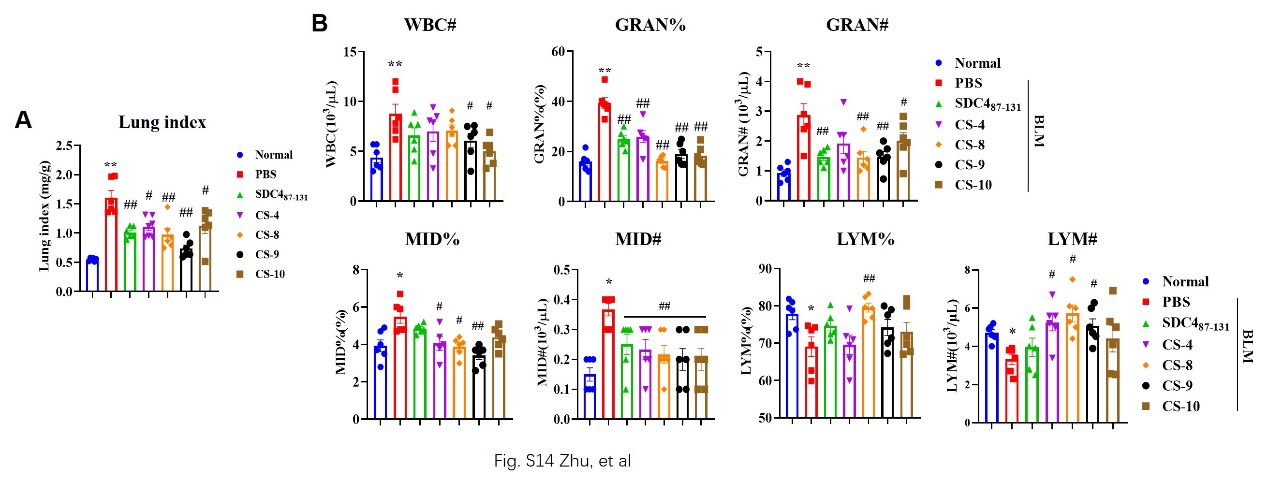


**Figure S15. Effects of designed peptides on lung index and blood cells.**

1. The lung index was calculated and compared (n=6); (**B**) Blood cells were counted using an animal blood analyzer (URIT BH-40Vct) (n=6). Note: *p < 0.05, **p < 0.01 vs. Normal group; #p < 0.05, ##p < 0.01 vs. PBS in BLM group; WBC: white blood cell; GRAN: neutrophil; MID: middle cell; LYM: lymphocyte.


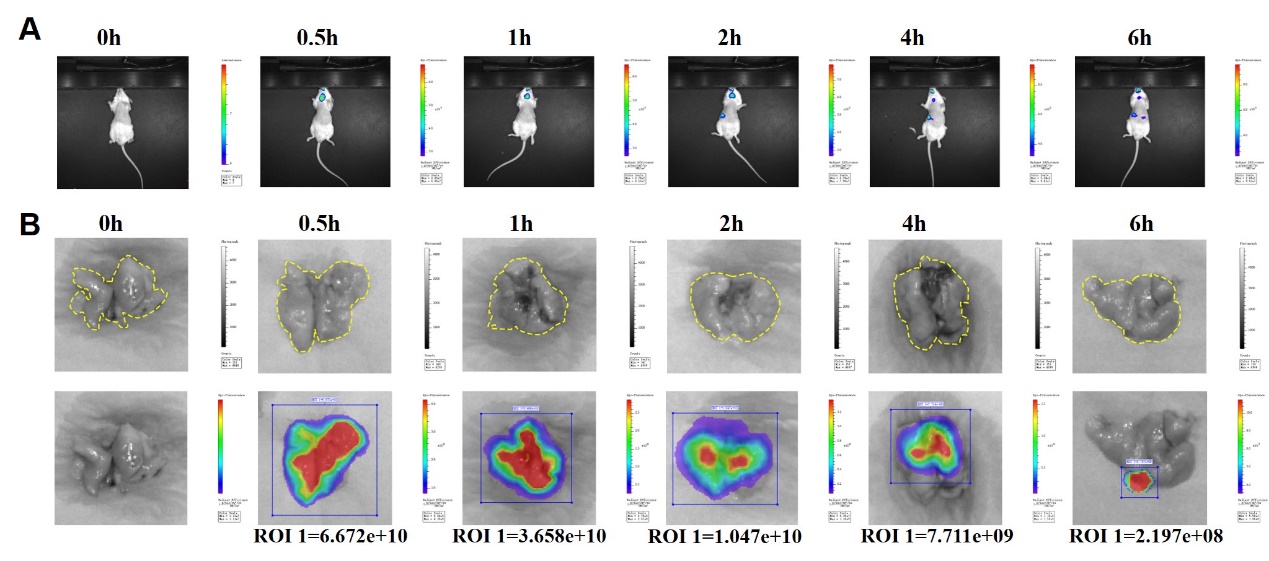


**Figure S16. Dynamic distribution of fluorescently labeled CS-9 in lung tissue**

CS-9 was fluorescently labeled with Cy5.5-NHS ester and administered intratracheally. Subsequent fluorescence imaging was performed using the PerkinElmer IVIS SPECTRUM In Vivo Imaging System. (**A**) In vivo imaging at various time points demonstrated no detectable pulmonary fluorescence signals due to the anatomical position of lung tissue within the thoracic cavity. (**B**) Ex vivo fluorescence imaging of lung tissues with ROI-based fluorescence intensity analysis revealed peak fluorescence intensity with homogeneous distribution throughout lung tissue at 0.5 hours post-administration, followed by a gradual decrease in fluorescence intensity and a reduction in the distribution area over time.
